# CMASA: an accurate algorithm for detecting local protein structural similarity and its application to enzyme catalytic site annotation

**DOI:** 10.1186/1471-2105-11-439

**Published:** 2010-08-27

**Authors:** Gong-Hua Li, Jing-Fei Huang

**Affiliations:** 1 State Key Laboratory of Genetic Resources and Evolution, Kunming Institute of Zoology, Chinese Academy of Sciences, 32, Eastern Jiaochang Road, Kunming, Yunnan 650223, China; 2 Graduate School of Chinese Academy of Sciences, Beijing 100039, China; 3 Kunming Institute of Zoology-Chinese University of Hongkong Joint Research Center for Bio-resources and Human Disease Mechanisms, Kunming 650223, China

## Abstract

**Background:**

The rapid development of structural genomics has resulted in many "unknown function" proteins being deposited in Protein Data Bank (PDB), thus, the functional prediction of these proteins has become a challenge for structural bioinformatics. Several sequence-based and structure-based methods have been developed to predict protein function, but these methods need to be improved further, such as, enhancing the accuracy, sensitivity, and the computational speed. Here, an accurate algorithm, the CMASA (Contact MAtrix based local Structural Alignment algorithm), has been developed to predict unknown functions of proteins based on the local protein structural similarity. This algorithm has been evaluated by building a test set including 164 enzyme families, and also been compared to other methods.

**Results:**

The evaluation of CMASA shows that the CMASA is highly accurate (0.96), sensitive (0.86), and fast enough to be used in the large-scale functional annotation. Comparing to both sequence-based and global structure-based methods, not only the CMASA can find remote homologous proteins, but also can find the active site convergence. Comparing to other local structure comparison-based methods, the CMASA can obtain the better performance than both FFF (a method using geometry to predict protein function) and SPASM (a local structure alignment method); and the CMASA is more sensitive than PINTS and is more accurate than JESS (both are local structure alignment methods). The CMASA was applied to annotate the enzyme catalytic sites of the non-redundant PDB, and at least 166 putative catalytic sites have been suggested, these sites can not be observed by the Catalytic Site Atlas (CSA).

**Conclusions:**

The CMASA is an accurate algorithm for detecting local protein structural similarity, and it holds several advantages in predicting enzyme active sites. The CMASA can be used in large-scale enzyme active site annotation. The CMASA can be available by the mail-based server (http://159.226.149.45/other1/CMASA/CMASA.htm).

## Background

With the development of both the genome project and the structural genomics, large of unknown functional protein structures were deposited in PDB, these protein functions need to be annotated. In addition, because of the fast development of bioinformatics, some known structure and function proteins may also need to be re-annotated. Thus, several methods of protein structural and functional prediction were developed, which can be classified as the sequence-based and the structure-based methods.

Sequence-based methods, such as, BLAST/PSI-BLAST[1,2] or PROSITE[3], are based on the concept of "similar protein sequences with similar function". The performance of these methods critically depends on the sequence similarity between the query structure and annotated structure. However, these methods may fail to detect the remote homologous and convergent proteins. In addition, the changes of some key residues may also result in the change of protein function, even though their sequence identities are very high. For example, VRK3, a member of kinase family, have lost its function as kinase and become into regulating other kinase activity, because the key ATP binding sites were mutated [4]. Thus, sequence-based methods may also fail to annotate the functional diversified proteins.

Structure-based methods contain the global and local structure comparison methods. Though the global structure-based methods, such as DALI [5], VAST [6], SSM [7] and CE [8], can detect the remote homologue proteins, they fail to detect the functional convergence of some proteins with different fold. For example, the enzymes with different folds, the trypsins and subtilisins, can hold same function of hydrolysis [9], but the global structure comparisons can not detect them each other. Some proteins with similar structures can perform different functions [10], but the global structure-based methods can not detect the functional divergence of some proteins with same fold.

The local structure-based methods can detect the functional convergence and predict the functional sites for those proteins with the less annotated structures, for example, FFF[11], PINTS[12], SPASM[13], JESS[14], Query3d [15], ASSAM [16], Cavbase[17], ef-Site [18] and so on. The FFF can search local structural similarities by the local structural geometry characters and the contact matrix constraint by user predefined, which has been successfully applied in predicting the active sites of glutaredoxins/thioredoxins and T1 ribonucleases[11]. Other methods search local structural similarities by the recursive enumeration strategy[19]. The core of this strategy is to extend initial candidate solutions[14,19]. Thus, the performances of these methods depend critically on the constraints that can extend the partial candidate solution quickly and accurately [14]. As the constraint, the Max Inter-Distance Deviation (MIDD) is well applied in most of the local structure comparison algorithms [12,13]. The results of these algorithms are sorted by the RMSD or the RMSD based P-value [12-14,20]. However, there is no restricting relationship between the MIDD and the RMSD. So the MIDD may be larger, but the RMSD may be very small in values. To obtain the better sensitivity and accuracy, the users have to define a larger MIDD, but the CPU time will increase dramatically. Thus, it is very difficult to balance the time cost and the performance.

In order to improve the local structure-based methods, the CMASA (Contact MAtrix based local Structural Alignment algorithm) has been used. The design requirements of CMASA are, as follows, (i) it should be not only fast but also sensitive and accurate enough for the large-scale structural annotation; (ii). It should be flexible enough for the different applications.

**Figure 1:**
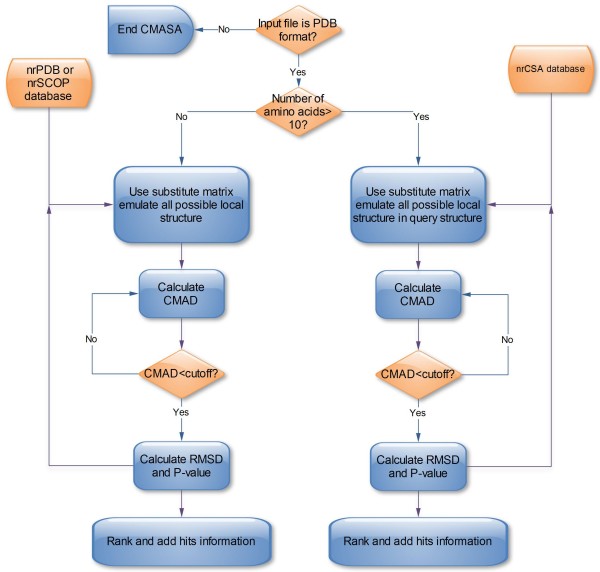
**Flowchart of CMASA algorithm**. The CMASA first decides that the query searches to the nrCSA database or the nrPDB/the nrSCOP database. Next, for each structure in database, the CMASA emulated all possible local structures using amino acid substitute. Again, the CMASA calculated the Contact Matrix Average Deviation (CMAD) between the active sites and possible local structures. Then, the CMASA calculated the RMSD and the RMSD based P-value if the CMAD < cutoff. At last, the CMASA ranked all of the hits and add their information.

To fulfil the above requirements, the steps to detect the local structure similarity in CMASA are shown in Figure 1. (i) Each residue in the protein structure is represented by both Ca (alpha-carbon atom) and Fa (furthest atom from alpha-carbon atom). (ii). Emulate all possible local structures that may match with the query structure by the residue substitute matrix, such as, Blosum62 [2]. (iii). Most of the possible local structures are discarded by the Contact Matrix Average Deviation (CMAD) rather than the MIDD. (iv). The RMSD is calculated by the Nelder-Mead Simplex Method[21]. (v). The RMSD-based p-value[20] and rank are computed.

Three CMASA's databases have been generated for different applications, 1) nrCSA from catalytic sites atlas (CSA)[22] for predicting enzyme active sites, 2) nrPDB and nrSCOP database for detecting remote homologues and convergent cases.

## Results

### Overview of the CMASA

The CMASA for detecting the local structure similarity can have different applications, when different databases are used. 1) The putative functional prediction of structured proteins by active sites database (now only nrCSA database is available). For example, the structure of 2qjw has been deposited by Joint Center for Structural Genomics (JCSG), but its function is unknown yet. When the PSI-BlAST is used, the 2qjw can hit nothing in PDB database and tens of hypothetical proteins in non-redundant (NR) database (p < 0.0005). These results suggest that 2qjw is a protein with unknown function. When the CMASA is used, the 2qjw can hit the 1a88, a haloperoxidase with the p-value of 6.7 × 10^-10^. And the 2qjw active sites predicted by the CMASA are S81, D129 and H155, which are same as the 1a88. Thus, the 2qjw may have haloperoxidase function. 2) The same catalytic sites in non-homologous proteins caused by convergent evolution can be observed by the nrPDB or nrSCOP. For example, the catalytic sites of 1djz (EC number: EC3.1.4.11) are H311, E431 and H356, 1djz catalytic sites can hit 2ddr (P-value = 0.02) with the EC number of EC3.1.4.12 by the CMASA, and the H311, E431 and H356 in 1djz are corresponding to the H296, D253 and H151 in the 2ddr. Thus, the results suggest that the both 1djz and 2ddr should hold same transformational reaction. Actually, both H296 and D253 are the catalytic residues in 2ddr[23], although 1djz and 2ddr hold different folds, where 1djz is belonged to TIM beta/alpha-barrel fold, but 2ddr is belonged to DNase I-like fold[24]. Thus, the transformational mechanism between 1djz and 2ddr may be resulted from convergent evolution.

**Figure 2:**
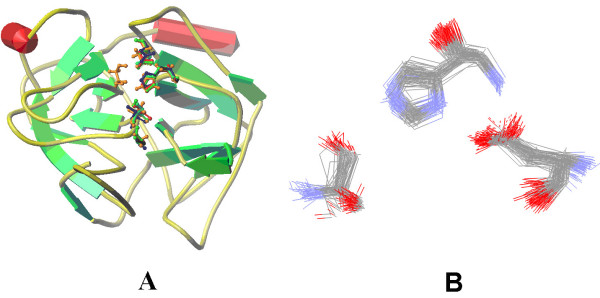
**The superposition output of the CMASA**. A: a structure (pdbid:1mct) searched to a functional sites database (nrCSA); highest five ranks were shown. The matched active sites were labelled as ball and stick. B: a functional site (1mct catalytic site: H57, D102 and S195) searched to the nrPDB; the hits with P-value < 1.0 × 10^-4 ^were shown (83 hits).

There are two kinds of output files after searching finished. One is the plain text file or html file(link to PDBsum[25]) similar as the BLAST output [2], in which the results are ranked by P-value. Another is a structure superposition file, where the hits below the superposition-cutoff value are superimposed. The superposition results of the protein (1mct) searching against the nrCSA database are shown in the Figure 2A, and the results of the active sites of 1mct (H57, D102, S195) searching against the nrPDB are shown in the Figure 2B.

The running speed of CMASA is also fast. When CMASA was running on the personal computer with the Intel Core 2 Duo E8400 3.0 GHz CPU, a protein with 400 residues is as the seed to search against the nrCSA database (1320 templates) by the defeat settings, the time cost is about 6 s (seconds). When the active sites including 3 residues are used to search against the nrSCOP (14541 structures) by the defeat settings, the time cost is about 30~60 s. The CMASA mail-based server (http://159.226.149.45/other1/CMASA/CMASA.htm) will reply the mails and give the searching results within 3 minutes if the server is not too busy.

### Constraint analysis of CMASA

**Figure 3:**
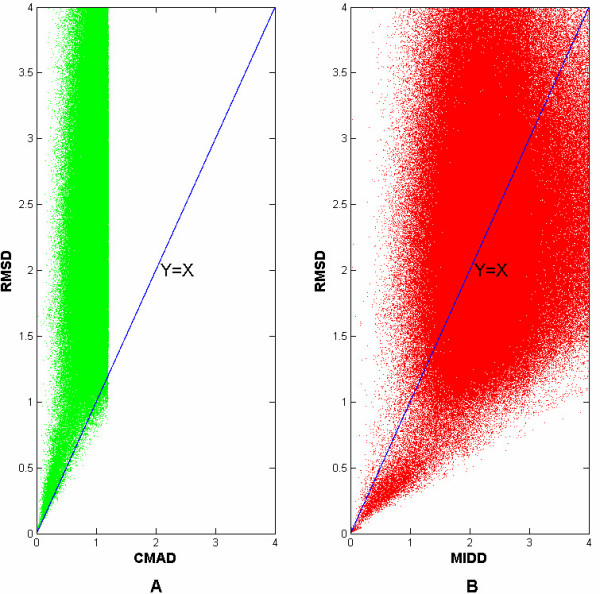
**The relationship between CMAD, MIDD and RMSD**. A: relationship between the CMAD and the RMSD. Each dot represents a pair of the CMAD and the RMSD. The relationship was only showed when the CMAD< = 1.2Å, because the CMAD cutoff was set as 1.2Å. The green line represents the line of Y = X. B: relationship between the MIDD and the RMSD, The green line also represents the line of Y = X.

A suitable constraint is very important for local structural alignment. For example, there are 135 candidates for emulating all H-D-S possible active sites in 1mct, a member of trypsin. The RMSD will have to be calculated to 135 times, if there is not any constraint. However, if we set the constraints of CMAD (Contact Matrix Average Deviation) <1.2 Å, the RMSD will be calculated just twice. As mentioned above, the CMAD has been used as the constraint in the CMASA rather the MIDD used in other methods. In theory, CMAD< = 2RMSD, but if the numbers of atoms is just two, CMAD = 2RMSD. In fact, there are at least 6 atoms, that is, 3 Ca and 3 Fa atoms. To access the exact relationship between the CMAD and the RMSD, we searched all the nrPDB data against the CSA database and plot each pair of CMAD and RMSD by the threshold of CMAD < 1.2 Å. The result (596477 pairs) shows that almost all the CMAD smaller than the RMSD with the P-value(CMAD< = RMSD) = 0.997(Figure 3A). However, as showed in Figure 3B, the relationship between the RMSD and the MIDD is complex, and there is no theoretical relationship between the RMSD and the MIDD. So, the CMAD is a suitable constraint for CMASA search. In practice, 1.2 Å as the threshold is enough for the local structural searching.

### Sensitivity and Accuracy of CMASA

**Figure 4:**
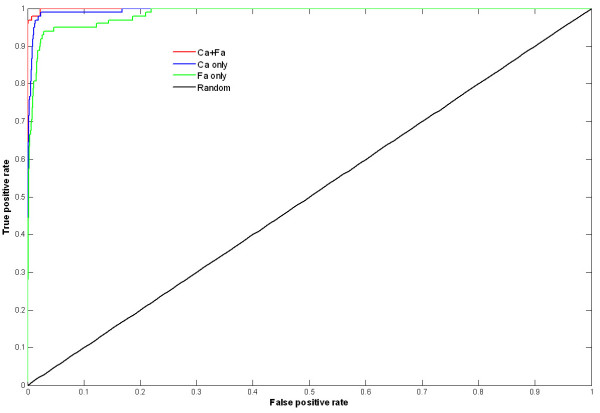
**ROC curves of Ca only, Fa only and combining both Ca and Fa**. The ROC curves were generated by 1mct active site (H57, D102 and S195) querying to nrSCOP using CMASA. The CMASA hits were ranked by P-value. The totally positives was 100 (85 trypsins and 15 subtilisins), and the totally negatives was 14441 (14541 nrSCOP minus 100 positives).

To obtain a better performance, different amino acid presentations were compared. The trypsin-like serine proteases superfamily and subtilisin-like superfamily have been as the example to evaluate the performance of three different amino acid presentations: Ca atom only, Fa atom only and combining both Ca and Fa atoms. Trypsins and subtilisins are with different folds, but they hold the same catalytic sites and similar function. There are 122 trypsins and subtilisins in nrSCOP database, in which 101 are trypsins and 21 are subtilisins. However, the catalytic sites in 22 enzymes of 122 trypsins and subtilisins have been mutated, such as, the active sites, H41 and S175, have been mutated to S41 and G175 in 1a7s. Thus, there are totally 100 positives (85 trypsins and 15 subtlilisins). The catalytic sites (H57, D102 and S195) of 1mct (a typsin) were used as the query to search against nrSCOP database, and the Receiver Operating Characteristics (ROC) curve[26] (Figure 4) is obtained. The result shows that the presentation of combining both Ca and Fa atoms is the best performance in these three presentations. Thus, the presentation of combining both Ca and Fa atoms is used in the CMASA.

164 CSA families were selected to test the overall sensitivity and accuracy of the CMASA (Additional file 1). For each family, two different templates were chosen to search the training set, which contains both family members' structures and a constant negative dataset. One is the master template, and another is the mean conformational template (MCT). For each template, the negative dataset is a subset of the nrPDB(10582 structures), where the nrCSA and nrEC have been excluded.

**Figure 5:**
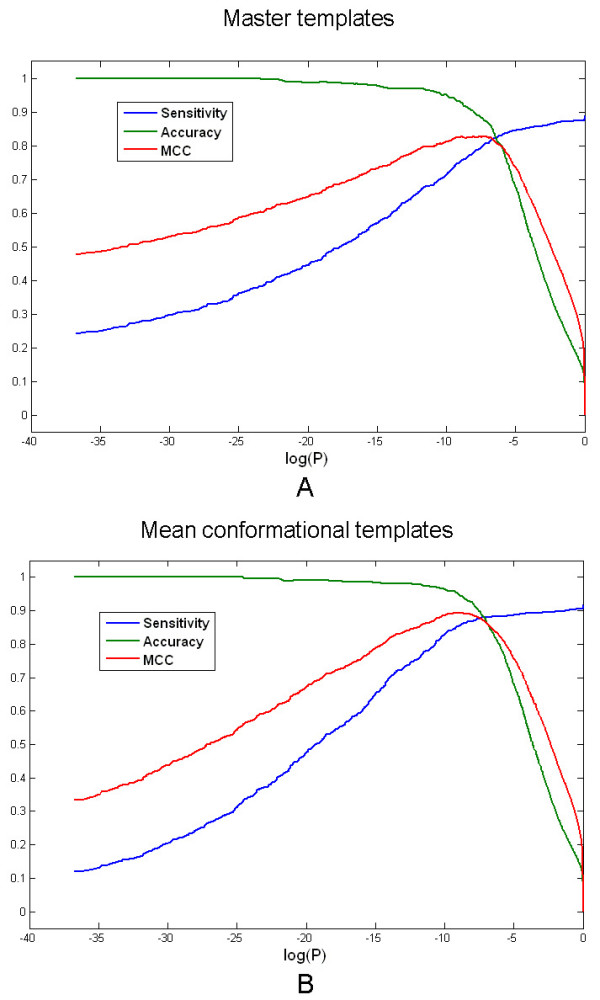
**The overall sensitivity, accuracy and MCC of the CMASA**. A. Using the master template. B. Using the mean template.

Several methods have been evaluated by ROC curve[26], but the overall performance of CMASA is evaluated by the Matthews Correlation Coefficient (MCC)[27], because MCC can clearly give the optimal threshold, which can suggest that the hit be true or false positive in large-scale active site annotation. The result (Figure 5) shows that the P-value threshold is 1.0 × 10^-4 ^for both master templates and mean conformational templates. We also calculated the RMSD based MCC (data not show), and got 0.85Å as the RMSD threshold for master templates and 0.84Å for mean conformational templates.

The MCC, sensitivity and accuracy of each family (Additional file 2) were calculated by using the optimal threshold. Because different protein homologous families have different MCC, sensitivity and accuracy, each data set was averaged. Table 1 shows the mean MCC, mean sensitivity and mean accuracy for all the 164 families in different template types and in different threshold types. The mean MCC is 0.90 with the mean sensitivity of 0.86 and with the mean accuracy of 0.96 by using mean conformational templates and P-value threshold. When the RMSD threshold is used, the mean MCC and sensitivity decreased about 0.03. When mean conformational templates instead of master templates are used, the mean MCC and sensitivity can be increased about 0.09. All the mean accuracy is above 0.94 (Table 1).

**Table 1:** The table of mean MCC, sensitivity and accuracy

Template type	Threshold type	Threshold level	Mean MCC	Mean Sensitivity	Mean Accuracy
Mean conformational template	p-value	1.00E-04	0.90(0.19)	0.86(0.18)	**0.96**(0.12)
	RMSD	0.84Å	0.88(0.13)	0.83(0.20)	**0.96**(0.14)
Master template	p-value	1.00E-04	0.82(0.17)	0.75(0.25)	**0.94**(0.14)
	RMSD	0.85Å	0.79(0.18)	0.71(0.27)	**0.95**(0.14)

The mean MCC, mean sensitivity and mean accuracy of 164 families in different threshold type and in different template type. Values in brackets are standard deviation.

### Comparison of CMASA with both sequence-based and global structure-based methods

**Figure 6:**
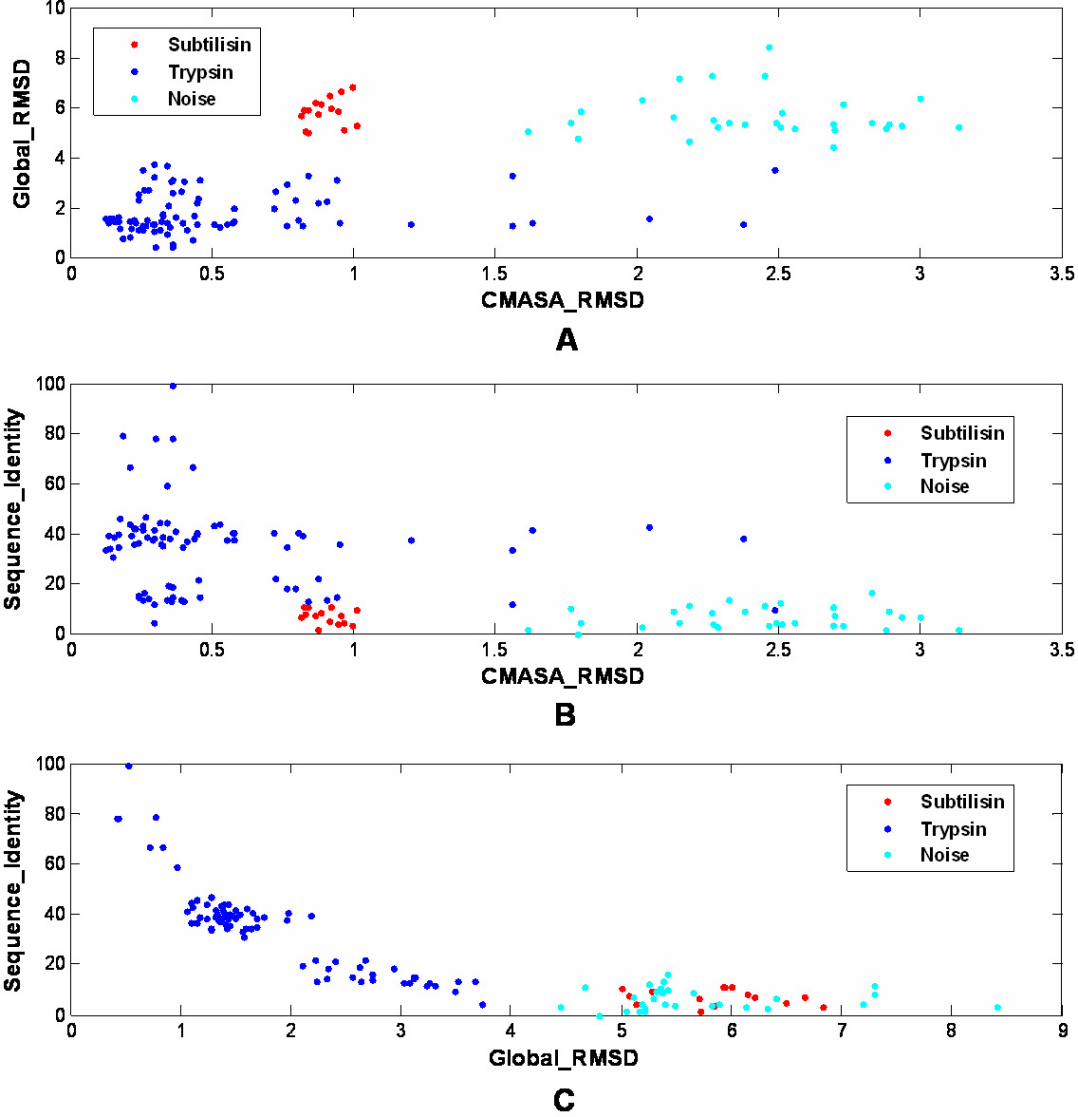
**CMASA compare with sequence-based, global structure-based methods**. A: The relationship between the CMASA RMSD and the global RMSD. B: The relationship between the CMASA RMSD and the sequence identity. C: The relationship between global RMSD and the sequence identity. 85 trypsins, 15 subtilisins and 30 random structures from the nrSCOP were selected for comparison. The CMASA_RMSD was calculated between 1mct active site (H57, D102 and S195) and the hits by the CMASA. The global_RMSD was calculated by CE package[8]. Sequence identity was calculated from structure-based sequence alignment.

The CMASA, sequence-based and global structure-based methods were compared by using the both trypsin-like serine proteases and subtilisin-like superfamilies. Figure 6 shows the relationships between the CMASA RMSD, the global RMSD (calculated by CE[8]) and the sequence identities. The results show that the CMASA can hit both trypsins and subtilisins from noise, even though their sequence and global structure similarities (Figure 6A and 6B) are low. The global structure-based method can hit all trypsins from noise, but it can not distinguish subtilisins from noise (Figure 6A and 6C). The results suggest that the global structure-based method is powerful for detecting the global structural similarity, but it is weak for detecting the local structural similarity; and the sequence-based method can only hit most of trypsins (Figure 6B and 6C), which can not distinguish some of remote homologous trypsins and subtilisins from noise. Thus, the CMASA can effectively find remote homologous proteins and the active site convergence comparing to the sequence-based method or to the global structure-based methods.

### Comparison between CMASA and other local structure comparing methods

Some local structural comparing methods have been applied in the enzyme active site annotation, such as, FFF[11], SPASM[13], PINTS[12], Query3d[15] and JESS[14]. The different residue representations and different searchable databases are used in these five methods above. For example, FFF only used the Ca atom, JESS used both Ca and Cb (beta carbon) atoms, SPASM used both Ca and a pseudo atom that is the geometrical centre of the residue. However, different methods have different searchable databases. Thus, it is difficult to compare them in overall. So we used some examples to evaluate the advantages and disadvantages between these five methods and the CMASA.

**Figure 7:**
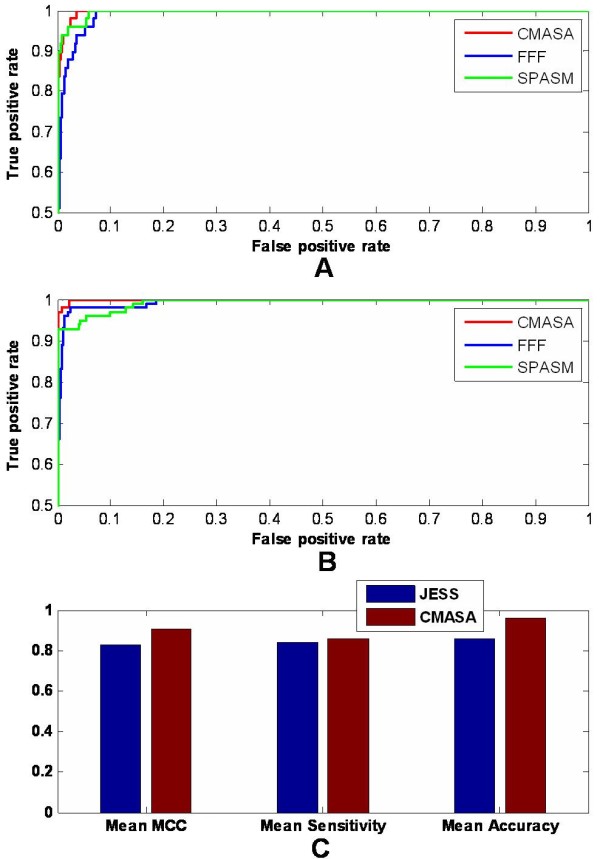
**CMASA compare with FFF, SPASM and JESS**. A. The ROC curve of the CMASA, the FFF and the SPASM using the 1aaz active site (C14, C17 and P66) searching to the nrSCOP. B. The ROC curve of the CMASA, the FFF and the SPASM using the 1mct active site (H57, D102 and S195) searching to the nrSCOP. C. The overall performance between the CMASA and the JESS.

Two cases have been used to compare the performance among the FFF, SPASM and CMASA. One is to recognise glutaredoxins/thioredoxins by 1aaz (a glutaredoxin) active residues (C14, C17 and P66). In nrSCOP database (14541 structures), there are 49 glutaredoxins/thioredoxins which have CxxC and P motif. Because the FFF only used geometry to predict protein function and did not calculate the RMSD or other scores for ranking, we ranked the FFF matches by the RMSD based P-value to compare to the CMASA. The SPASM matches are ranked by the RMSD. Then, the ROC curve is obtained (Figure 7A). The results show that the CMASA is better than the SPASM, and the SPASM is better than the FFF. Another case is to find trypsins and subtilisins by 1mct active sites (Figure 7B). The ROC curve shows that all of these three methods can hold the good performance, when they are used to detect the 1mct active site similarities (Figure 7B), but some differences can be also observed. The CMASA is remarkably better than the both SPASM and FFF, but the performance between the SPASM and the FFF is complex. When the false positive rate is small than 0.01, the SPASM can hit more true positives than the FFF. But when the false positive rate is larger than 0.01, the FFF can hit more true positives than the SPASM. These cases suggest that the performance difference between the SPASM and the FFF is complex, but the CMASA can get a better performance than the both SPASM and FFF.

PINTS have its own website, so we used 2ity (a protein kinase) active sites to search PINTS SCOP_specials database (a database of PINTS, same as nrSCOP database in this work). Interestingly, only 1 kinase can be hit and the best hit is not kinase. On the contrast, the CMASA can hit 20 kinases with the false positive rate <0.3. We also used the active sites of 1mct to search PINTS SCOP_specials database (SCOP version 1.61), 4 of 54 trypsins and 4 of 10 subtilisins can be hit. But 85 of 101 trypsins and 15 of 21 subtilisins can be hit by the CMASA. Thus, the sensitivity of CMASA is better than that of PINTS.

The Query3d is powerful to find similar local structures within two proteins. But it may be weak to detect the similarity between an active site and a protein. For example, we used 1k2p (a protein kinase) active sites to search PDB database in the Qurey3d website, not any hit is shown. However, the CMASA can give more than 20 positives (P-value < 0.01) and the reasonable rank. Thus, the Query3d may be not suitable for enzyme catalytic site annotation.

The JESS had used CSA families to evaluated the algorithm performance[28]. So the overall performance between the CMASA and the JESS were compared. The results had showed that the JESS had the maximum mean MCC of 0.83 with the mean sensitivity of 0.86 and the mean accuracy of 0.84 [28]. However, the CMASA can hold the mean MCC of 0.90 with the mean sensitivity of 0.86 and the mean accuracy of 0.96. Thus, the accuracy of the CMASA is higher than that of the JESS (Figure 7C).

### Large scale annotation of enzyme catalytic sites

The above results suggest that the sensitivity and accuracy of the CMASA may be enough for doing the large scale functional annotation. So the CMASA is also tried to annotate the enzyme catalytic sites. All the proteins in nrPDB were searched against the mean conformational template (MCT) dataset (1320 templates) by the CMASA, and 263 structures has been characterized, which are not annotated by the CSA2.2.9 (P-value < 1.0 × 10^-4^). In fact, 166 of 263 have been deposited before 2008 (Additional file 3). Thus, these results demonstrate that at least 166 putative novel catalytic sites can not be annotated by CSA (Additional file 3).

### Cases of enzyme catalytic site prediction

Two cases were used to evaluate the CMASA advantages further. The structure of 3BDV is from the Joint Center for Structural Genomics (JCSG) that aims to develop high-throughput methods for protein production, crystallization, and structure determination. 3BDV is belonged to UDF1234 family with unknown function[29]. 3BDV can hit 1JKM of a serine hydrolase (P-value = 3.40 × 10^-5^) by the CMASA, and the catalytic residues of 3BDV are predicted as S81, D135 and H162. Further sequence analysis shows that the whole UDF1234 family members are conserved in the sites of S81, D135 and H162 (Additional file 4), which suggest that the entire UDF1234 family members probably have a function similar as serine hydrolase with S-D-H active sites.

The catalytic sites of an arylsulfatase (PDBid: 1HDH) have been annotated in the CSA[22]. However, the catalytic sites of its one homologue (PDBid: 1P49) can not be found in the CSA. The PSI-BLAST result suggested that the 1P49 catalytic sites mismatch result fails to be annotated, because of the low sequence identity (Additional file 5). However, 1P49 can hit 1HDH with high confidence (P-value = 3.5 × 10^-13^) by the CMASA, and the catalytic residues are predicted as R79, K134, H136, H290, D342 and K368 (Additional file 5). The structural information [30] convinces this prediction.

## Discussion

An accurate algorithm, the CMASA, has been developed to detect the local protein structural similarity, which can not only search the similar functional proteins by query the active sites, but also predict an unknown protein function, including distant homologous proteins or convergent proteins, by searching to functional active site database.

When the CMAD is used as the constraint and the Ca/Fa atoms are used to represent the residues, the balance between sensitivity, accuracy and the time cost can be reached. The CMASA is fast by testing on PC, and maintains sensitive and highly accurate (>0.94) for searching enzyme active sites. So, the CMASA may be helpful for improving the large scale annotation.

The CMASA has been compared to other methods. These methods contain the sequence-based, the global structure-based and five local structure-based methods. The results suggest that the CMASA can get better performance than all of these methods in detecting enzyme active site similarity. PSI-BLAST[2] has been used to annotate the enzyme catalytic sites[22], but it is weak at annotating distant homologous proteins and convergent proteins. So the CMASA is an effective method to annotate the distant homologous or convergent protein/enzyme active sites.

Of course, some limitations can be found in the CMASA, for example, i) the protein structures are required; ii) the structural difference of the side chains between the query and hit active residues will affect the sensitivity.

## Conclusions

The CMASA is not only highly accurate but also sensitive and fast for detecting the local protein structural similarity. It can be applied in annotating the distant homologous or convergent protein/enzyme active sites. And at least putative 166 novel catalytic sites have been suggested by the CMASA. A mail-based server has been available.

## Methods

### Residue representation

To insure the accuracy and reduce the complexity, CMASA used all amino acids of the structures and each residue is represented by both Ca (alpha-carbon atom) and Fa (furthest atom from Ca). In addition, the only Ca and only Fa are also used to evaluate how well these two terms in combination provides more predictive performance.

### Search algorithm

The flowchart of CMASA was showed in Figure 1. First, the CMASA parsed the query and decided whether the query search the nrPDB/the nrSCOP or the nrCSA database. Second, the CMASA used substitute matrix to emulate all candidate matches. Third, the CMASA used Contact Matrix Average Deviation (CMAD) to filter the candidates. Forth, the RMSD and the RMSD based P-value are calculated to score the matches. Fifth, ranking the matches

### Constraint

The CMAD (Contact Matrix Average Deviation) is used as the constraint. Given template T(t1, t2,...., tn) and the possible match T'(t'1, t'2,....t'n), then:

(1) $$CMAD=\frac{1}{n*(n-1)}\sum_{i=1}^{n}\sum_{j=1}^{n}\left|d(t_{i},t_{j})-d(t_{i}^{'}-t_{j}^{'})\right|$$

Where *d*(*t_i_*,*t_j_*) is the distance between the atom i and atom j, and n is the number of atoms in the template. In fact, to get more convenience, the CMAD is calculated, as follows,

(2) CMAD=(CMAD(Ca)+CMAD(Fa))/2

### Calculation of the RMSD

We presented an algorithm to calculate the RMSD. Generally, calculating the RMSD is to find the R and R0 to minimize the RMSD.

(3) $$RMSD=\sqrt{\frac{\sum_{i=1}^{n}{\left(x1_{i}-x2_{i}^{'}\right)}^{2}+{\left(y1_{i}-y2_{i}^{'}\right)}^{2}+{\left(z1_{i}-z2_{i}^{'}\right)}^{2}}{n}}$$

(4) (x2',y2',z2')=(x2,y2,z2)*R+R0

Where R is the rotation matrix and R0 is the translation matrix. Here, we use Nelder-Mead Simplex Method[21] to solve this problem. This method uses the concept of a simplex, which is a special polytope of *N *+ 1 vertex in *N *dimensions, and is commonly used nonlinear optimization. The rotation matrix R is equal to Rx(α)* Ry(β)*Rz(γ)* Ry(β)^T^* Rx(α)^T^. Because the CMASA only superposes less than 10 amino acids, and because the geometric centre of two similar local structures should be superposited, the R0 can be pre-calculated through query geometric centre minus the match geometric centre. Therefore, the dimension of the simplex is N = 3. Then, using the Nelder-Mead Simplex minimization function in the GSL(GNU Scientific Library, which is a free numerical library for C and C++ programmers) or "fminsearch" function in Matlab/Octave (a software for computation and engineering), RMSD can be calculated.

### Statistic significance

The statistical significance score was calculated using the method of Stark *et al*[20], which was used in the PINTS web server[12].

(5) $$P(RMSD\leq R_{M})=1-e^{-EF(R_{M})}$$

(6) $$EF(R_{M})=a\Phi b^{N}R_{M}^{4.93N-5.88}$$

Where EF is expected number of matches with the RMSD or better, R_M _is the RMSD. N is the total number of query residues, Φ is the product of the percentage abundances of all residues. a and b are empirically constants: a = 473, b = 0.4.

### CMASA database

The nrPDB(non-redundant PDB, 18757 structures) was directly from PDB[31] (Version released on the 01-AUG-2008). All protein chains of at least 20 amino acids were clustered by blastclust (included in the BLAST[2] package) at 90% sequence similarity. Each cluster was ranked by structure resolution. The highest rank in each cluster was regarded as the represent structure. The overlap between the nrPDB and the pdbEC (http://www.ebi.ac.uk/thornton-srv/databases/pdbsum/data/pdb_EC) is the non-redundant pdbEC (5189 structures).

The nrSCOP (non-redundant structures from SCOP) is from the SCOP[24](Version 1.75). The SCOP database has 7 levels: root, class, fold, superfamily, family, protein and species. In each species level, only the first structure was selected. All of these selected structures formed the nrSCOP (14541 structures).

The nrCSA (non-redundant catalytic sites atlas) is from the catalytic sites atlas (CSA) with the version2.2.9[22]. Some CSA templates that only contain one or two residues are removed, because one amino acid means nothing for catalytic mechanism, and because only two amino acids will give too much noise in the CMASA results. Rather more, some CSA templates, which have 3 residues but 2 of them are glycines, are also removed. These "4 atoms" CSA templates (1 Fa and 3 Ca atoms) are similar as only two amino acid templates (also 4 atoms: 2 Fa and 2 Ca atoms), so these templates will also give too much noise in the CMASA results. The overlap between the nrPDB and the CSA is the nrCSA.

### Master templates and Mean conformational templates (MCT)

All nrCSA templates with the same EC number and the same active sites are grouped. For each group, the master template is defined as the one which makes the sumRMSD minimal, the sumRMSD is:

(7) $$sumRMSD(i)=\sum_{j\neq i}^{n}RMSD(i,j)$$

Where *RMSD*(*i*,*j*) is the RMSD between ith and jth template in the group; n is the number of the templates in the group.

The residue information of MCT is extracted from the master template, but the three-dimensional coordinates are changed, and they are:

(8) $${\left(x,y,z\right)}_{mct}=\frac{1}{m+1}({\left(x,y,z\right)}_{master}+\sum_{j=1}^{m}{\left(x',y',z'\right)}_{j})$$

Where (*x*',*y*',*z*'), means the three-dimensional coordinates of jth template which have superposed to the master template; m is the number of the templates with *RMSD*(*master*,*j*)≤1.5 Å.

### Sensitivity and specificity analysis

Two methods are used for evaluating the CMASA performance. One is the ROC curve[26], another is the Matthews correlation coefficient(MCC)[27]. The ROC curve is used for comparing the CMASA and other methods. The ROC curve is the plot of the true positives (Tp) rate and the false positives (Fp) rate.

The MCC method was used in overall sensitivity and accuracy analysis and used in calculating the overall optimal threshold. The MCC is calculated as:

(9) $$MCC=\frac{TpTn-FpFn}{\sqrt{\left(Tp+Fp)(Tp+Fn)(Tn+Fp)(Tn+Fn\right)}}$$

(10) $$Sensitivity=\frac{Tp}{Tp+Fn}$$

(11) $$Accuracy=\frac{Tp}{Tp+Fp}$$

Where Tp, Tn, Fp and Fn are the true positives, true negatives, false positives and false negatives, respectively.

164 CSA families are used for evaluating the CMASA overall performance. These families are generated by the following steps: 1) the nrCSA members with same EC number are grouped together. 2) In each group, these members with same active sites are grouped to a CSA family. These families with less than 3 members are discarded. As a result, we got 164 CSA families to analysis the sensitivity and specificity (Supplement Table S1). The negative data set (10582 structures) is a subset of the nrPDB, which is deposited before 2008 and excludes the nrCSA and enzymes.

For each 164 CSA families, both the master template and the mean conformational template are generated to query against a training set, which is the combination of the family members(positives) and a constant negative data set (10582 structures). All hits of 164 CSA families are combined and ranked by the P-value or the RMSD. So there are 1033 positives (sum of 164 families' positives) and 10582 negatives. Then, the overall MCC, sensitivity and accuracy are calculated (Figure 5A and 5B).

The overall optimal threshold is defined as RMSD or P-value where the overall MCC is at a maximum. After the overall optimal threshold is defined, the hits of each family, where the RMSD or the P-value is small than the overall optimal threshold, are used to calculate the MCC, sensitivity and accuracy in each family (Additional file 2).

## Authors' contributions

JFH directed the data analysis, method development, and writing of the manuscript. GHL performed the data analysis and method development, including programming, and wrote the manuscript. Both authors have read and approved this manuscript.

## Supplementary Material

Additional file 1 **Table S1: The CSA families and its active sites and family members**. Click here for file

Additional file 2 **Table S2: The MCC, Sensitivity and specificity in different family using the overall threshold**. Click here for file

Additional file 3 **Table S3: Predicted active sites with P-value < 1.0 × 10^-4 ^and their best matching MCT CSA. Only showed the predicted structures deposited before 2008**. Click here for file

Additional file 4 **Figure S1: Predicting 3BDV catalytic sites using CMASA**. A: The CMASA superposition result. The best hit, a serine hydrolase (PDBid:1JKM) with the catalytic sites of S202-D303-H338, is shown. The predicted 3BDV catalytic sites (S81, D135 and H162) are labelled. B: the sequence alignment of the DUF123 family, these sequences are directly from Pfam[29] seed sequences. The predicted catalytic sites are labelled by inverted triangles. Click here for file

Additional file 5 **Figure S2: Predicting the catalytic sites of human placental estrone sulfatase (PDBid:**1P49) **using CMASA**. A: the CMASA superposition result. The best hit, an arylsulfatase (PDBid: 1HDH), which hold the catalytic sites of R55-K113-H115-H211-D317-K375, is shown. The predicted 1P49 catalytic sites (R79, K134, H136, H290, D342 and K368) are labelled. B: PSI-BLAST result between 1P49 and 1HDH. The predicted 1P49 catalytic sites and the 1HDH catalytic sites are labelled as inverted red and blue triangles. Click here for file
