# Supplementary material for: CMASA: an accurate algorithm for detecting local protein structural similarity and its application to enzyme catalytic site annotation
Source: BMC Bioinformatics. 2010 Aug 27;11:439. doi: 10.1186/1471-2105-11-439 (PMC2936402; doi:10.1186/1471-2105-11-439)
Supplement: Additional file 2 — Table S2: The MCC, Sensitivity and specificity in different family using the overall threshold. [file 1471-2105-11-439-S2.DOC]

| **Family name** | **RMSD threshold** | | | | | | **P-value threshold(1.0e-04)** | | | | | |
| --- | --- | --- | --- | --- | --- | --- | --- | --- | --- | --- | --- | --- |
|  | **Master template(0.85)** | | | **mean conformational template(0.84)** | | | **master template** | | | **mean template** | | |
|  | mcc | sens | specific | mcc | sens | specific | mcc | sens | specific | mcc | sens | specific |
| 1A8IA | 0.63 | 0.40 | 1.00 | 0.77 | 0.60 | 1.00 | 0.63 | 0.40 | 1.00 | 0.77 | 0.60 | 1.00 |
| 1ACOA | 1.00 | 1.00 | 1.00 | 1.00 | 1.00 | 1.00 | 0.71 | 1.00 | 0.50 | 1.00 | 1.00 | 1.00 |
| 1ADOA | 0.94 | 1.00 | 0.89 | 0.94 | 1.00 | 0.89 | 0.87 | 0.75 | 1.00 | 1.00 | 1.00 | 1.00 |
| 1AFWA | 0.87 | 0.75 | 1.00 | 0.87 | 0.75 | 1.00 | 0.87 | 0.75 | 1.00 | 0.87 | 0.75 | 1.00 |
| 1AJOA | 0.94 | 1.00 | 0.88 | 0.94 | 1.00 | 0.88 | 0.94 | 1.00 | 0.88 | 0.94 | 1.00 | 0.88 |
| 1AL6A | 0.82 | 0.67 | 1.00 | 0.82 | 0.67 | 1.00 | 0.82 | 0.67 | 1.00 | 0.82 | 0.67 | 1.00 |
| 1AQUA | 0.82 | 0.67 | 1.00 | 1.00 | 1.00 | 1.00 | 0.82 | 0.67 | 1.00 | 1.00 | 1.00 | 1.00 |
| 1AT1A | 0.71 | 0.50 | 1.00 | 0.87 | 0.75 | 1.00 | 0.87 | 0.75 | 1.00 | 1.00 | 1.00 | 1.00 |
| 1ATJA | 1.00 | 1.00 | 1.00 | 1.00 | 1.00 | 1.00 | 1.00 | 1.00 | 1.00 | 1.00 | 1.00 | 1.00 |
| 1AW5A | 1.00 | 1.00 | 1.00 | 1.00 | 1.00 | 1.00 | 1.00 | 1.00 | 1.00 | 1.00 | 1.00 | 1.00 |
| 1BDMA | 1.00 | 1.00 | 1.00 | 1.00 | 1.00 | 1.00 | 1.00 | 1.00 | 1.00 | 1.00 | 1.00 | 1.00 |
| 1BITA | 0.91 | 1.00 | 0.83 | 0.91 | 1.00 | 0.83 | 0.91 | 1.00 | 0.83 | 0.91 | 1.00 | 0.83 |
| 1BK7A | 1.00 | 1.00 | 1.00 | 1.00 | 1.00 | 1.00 | 0.71 | 0.50 | 1.00 | 0.87 | 0.75 | 1.00 |
| 1BMFD | 0.71 | 0.50 | 1.00 | 0.71 | 0.50 | 1.00 | 0.71 | 0.50 | 1.00 | 0.71 | 0.50 | 1.00 |
| 1BX4A | 0.50 | 0.25 | 1.00 | 0.71 | 0.50 | 1.00 | 0.50 | 0.25 | 1.00 | 0.71 | 0.50 | 1.00 |
| 1BZCA | 0.87 | 0.75 | 1.00 | 0.87 | 0.75 | 1.00 | 0.87 | 0.75 | 1.00 | 0.87 | 0.75 | 1.00 |
| 1C2BA | 0.82 | 1.00 | 0.67 | 0.76 | 1.00 | 0.57 | 0.75 | 0.75 | 0.75 | 0.89 | 1.00 | 0.80 |
| 1C9WA | 1.00 | 1.00 | 1.00 | 1.00 | 1.00 | 1.00 | 1.00 | 1.00 | 1.00 | 1.00 | 1.00 | 1.00 |
| 1CBGA | 0.82 | 0.67 | 1.00 | 1.00 | 1.00 | 1.00 | 0.82 | 0.67 | 1.00 | 0.82 | 0.67 | 1.00 |
| 1CBXA | 0.58 | 0.33 | 1.00 | 1.00 | 1.00 | 1.00 | 0.58 | 0.33 | 1.00 | 1.00 | 1.00 | 1.00 |
| 1CF2O | 0.67 | 0.67 | 0.67 | 1.00 | 1.00 | 1.00 | 0.87 | 1.00 | 0.75 | 1.00 | 1.00 | 1.00 |
| 1CGTA | 0.82 | 0.67 | 1.00 | 1.00 | 1.00 | 1.00 | 1.00 | 1.00 | 1.00 | 1.00 | 1.00 | 1.00 |
| 1CKIA | 0.71 | 0.50 | 1.00 | 0.82 | 0.67 | 1.00 | 0.82 | 0.67 | 1.00 | 0.82 | 0.67 | 1.00 |
| 1CLKA | 1.00 | 1.00 | 1.00 | 1.00 | 1.00 | 1.00 | 1.00 | 1.00 | 1.00 | 1.00 | 1.00 | 1.00 |
| 1CMVA | 0.82 | 0.67 | 1.00 | 1.00 | 1.00 | 1.00 | 0.82 | 0.67 | 1.00 | 1.00 | 1.00 | 1.00 |
| 1CMXA | 0.58 | 0.33 | 1.00 | 0.58 | 0.33 | 1.00 | 0.82 | 0.67 | 1.00 | 1.00 | 1.00 | 1.00 |
| 1CV2A | 1.00 | 1.00 | 1.00 | 1.00 | 1.00 | 1.00 | 1.00 | 1.00 | 1.00 | 1.00 | 1.00 | 1.00 |
| 1CVIA | 1.00 | 1.00 | 1.00 | 1.00 | 1.00 | 1.00 | 1.00 | 1.00 | 1.00 | 1.00 | 1.00 | 1.00 |
| 1CZFA | 0.91 | 0.83 | 1.00 | 0.91 | 0.83 | 1.00 | 0.91 | 0.83 | 1.00 | 1.00 | 1.00 | 1.00 |
| 1D3AA | 1.00 | 1.00 | 1.00 | 1.00 | 1.00 | 1.00 | 0.94 | 0.89 | 1.00 | 1.00 | 1.00 | 1.00 |
| 1D3GA | 1.00 | 1.00 | 1.00 | 1.00 | 1.00 | 1.00 | 1.00 | 1.00 | 1.00 | 1.00 | 1.00 | 1.00 |
| 1D6OA | 0.96 | 0.92 | 1.00 | 0.96 | 0.92 | 1.00 | 0.91 | 0.83 | 1.00 | 0.96 | 0.92 | 1.00 |
| 1DJLA | 0.50 | 0.25 | 1.00 | 0.71 | 0.50 | 1.00 | 0.50 | 0.25 | 1.00 | 0.71 | 0.50 | 1.00 |
| 1DL2A | 0.87 | 0.75 | 1.00 | 1.00 | 1.00 | 1.00 | 1.00 | 1.00 | 1.00 | 1.00 | 1.00 | 1.00 |
| 1DNPA | 1.00 | 1.00 | 1.00 | 1.00 | 1.00 | 1.00 | 1.00 | 1.00 | 1.00 | 1.00 | 1.00 | 1.00 |
| 1DV7A | 0.94 | 0.88 | 1.00 | 0.94 | 0.88 | 1.00 | 0.87 | 0.88 | 0.88 | 0.94 | 1.00 | 0.89 |
| 1DXLA | 1.00 | 1.00 | 1.00 | 1.00 | 1.00 | 1.00 | 1.00 | 1.00 | 1.00 | 1.00 | 1.00 | 1.00 |
| 1DYSA | 0.58 | 0.33 | 1.00 | 0.82 | 0.67 | 1.00 | 0.58 | 0.33 | 1.00 | 0.82 | 0.67 | 1.00 |
| 1DYWA | 0.84 | 0.71 | 1.00 | 0.91 | 0.82 | 1.00 | 0.94 | 0.89 | 1.00 | 0.94 | 0.89 | 1.00 |
| 1E1OA | 1.00 | 1.00 | 1.00 | 1.00 | 1.00 | 1.00 | 0.82 | 0.67 | 1.00 | 1.00 | 1.00 | 1.00 |
| 1E7PA | 0.45 | 0.20 | 1.00 | 0.77 | 0.60 | 1.00 | 0.63 | 0.40 | 1.00 | 0.77 | 0.60 | 1.00 |
| 1EC7A | 0.82 | 0.67 | 1.00 | 1.00 | 1.00 | 1.00 | 1.00 | 1.00 | 1.00 | 1.00 | 1.00 | 1.00 |
| 1ECLA | 0.82 | 0.67 | 1.00 | 0.82 | 0.67 | 1.00 | 0.82 | 0.67 | 1.00 | 1.00 | 1.00 | 1.00 |
| 1EDGA | 0.71 | 0.50 | 1.00 | 0.87 | 0.75 | 1.00 | 0.75 | 0.75 | 0.75 | 0.75 | 0.75 | 0.75 |
| 1EG1A | 0.87 | 1.00 | 0.75 | 0.87 | 1.00 | 0.75 | 0.87 | 1.00 | 0.75 | 0.87 | 1.00 | 0.75 |
| 1EH6A | 0.89 | 0.80 | 1.00 | 0.89 | 0.80 | 1.00 | 0.89 | 0.80 | 1.00 | 0.89 | 0.80 | 1.00 |
| 1EJJA | 0.82 | 0.67 | 1.00 | 0.82 | 0.67 | 1.00 | 0.82 | 0.67 | 1.00 | 0.82 | 0.67 | 1.00 |
| 1EMSA | 0.82 | 0.67 | 1.00 | 1.00 | 1.00 | 1.00 | 0.82 | 0.67 | 1.00 | 0.82 | 0.67 | 1.00 |
| 1F6DD | 0.71 | 0.50 | 1.00 | 0.71 | 0.50 | 1.00 | 0.71 | 0.50 | 1.00 | 0.71 | 0.50 | 1.00 |
| 1F75A | 0.50 | 0.25 | 1.00 | 0.71 | 0.50 | 1.00 | 0.50 | 0.25 | 1.00 | 0.71 | 0.50 | 1.00 |
| 1FCQA | 1.00 | 1.00 | 1.00 | 1.00 | 1.00 | 1.00 | 1.00 | 1.00 | 1.00 | 1.00 | 1.00 | 1.00 |
| 1FGXA | 0.82 | 0.67 | 1.00 | 0.82 | 0.67 | 1.00 | 0.58 | 0.33 | 1.00 | 0.82 | 0.67 | 1.00 |
| 1FJMA | 1.00 | 1.00 | 1.00 | 1.00 | 1.00 | 1.00 | 1.00 | 1.00 | 1.00 | 1.00 | 1.00 | 1.00 |
| 1FOBA | 0.89 | 1.00 | 0.80 | 0.89 | 1.00 | 0.80 | 0.89 | 1.00 | 0.80 | 0.89 | 1.00 | 0.80 |
| 1FUGA | 0.58 | 0.33 | 1.00 | 1.00 | 1.00 | 1.00 | 1.00 | 1.00 | 1.00 | 1.00 | 1.00 | 1.00 |
| 1FUOA | 0.58 | 0.33 | 1.00 | 0.82 | 0.67 | 1.00 | 0.82 | 0.67 | 1.00 | 0.82 | 0.67 | 1.00 |
| 1FUSA | 0.67 | 0.67 | 0.67 | 0.67 | 0.67 | 0.67 | 0.67 | 0.67 | 0.67 | 0.82 | 0.67 | 1.00 |
| 1FX0A | 0.58 | 0.33 | 1.00 | 0.82 | 0.67 | 1.00 | 0.58 | 0.33 | 1.00 | 0.82 | 0.67 | 1.00 |
| 1G0DA | 1.00 | 1.00 | 1.00 | 1.00 | 1.00 | 1.00 | 1.00 | 1.00 | 1.00 | 1.00 | 1.00 | 1.00 |
| 1G0ZA | 0.72 | 0.91 | 0.57 | 0.70 | 0.89 | 0.55 | 0.79 | 0.77 | 0.82 | 0.81 | 0.80 | 0.82 |
| 1G2OA | 0.82 | 0.67 | 1.00 | 0.82 | 0.67 | 1.00 | 0.71 | 0.50 | 1.00 | 0.82 | 0.67 | 1.00 |
| 1GALA | 1.00 | 1.00 | 1.00 | 1.00 | 1.00 | 1.00 | 1.00 | 1.00 | 1.00 | 1.00 | 1.00 | 1.00 |
| 1GEHA | 0.71 | 0.50 | 1.00 | 0.71 | 0.50 | 1.00 | 0.87 | 0.75 | 1.00 | 0.87 | 0.75 | 1.00 |
| 1GERA | 0.58 | 0.33 | 1.00 | 0.82 | 0.67 | 1.00 | 0.58 | 0.67 | 0.50 | 0.82 | 0.67 | 1.00 |
| 1GK8A | 0.87 | 0.75 | 1.00 | 0.87 | 0.75 | 1.00 | 0.87 | 0.75 | 1.00 | 1.00 | 1.00 | 1.00 |
| 1GOKA | 0.94 | 0.89 | 1.00 | 1.00 | 1.00 | 1.00 | 0.94 | 0.89 | 1.00 | 1.00 | 1.00 | 1.00 |
| 1GPIA | 0.87 | 1.00 | 0.75 | 0.87 | 1.00 | 0.75 | 0.87 | 1.00 | 0.75 | 0.87 | 1.00 | 0.75 |
| 1GQNA | 1.00 | 1.00 | 1.00 | 1.00 | 1.00 | 1.00 | 1.00 | 1.00 | 1.00 | 1.00 | 1.00 | 1.00 |
| 1GX3A | 0.85 | 1.00 | 0.71 | 0.85 | 1.00 | 0.71 | 0.85 | 1.00 | 0.71 | 0.85 | 1.00 | 0.71 |
| 1GZ7A | 0.77 | 1.00 | 0.60 | 0.71 | 1.00 | 0.50 | 0.87 | 1.00 | 0.75 | 0.87 | 1.00 | 0.75 |
| 1HVXA | 0.82 | 0.67 | 1.00 | 0.82 | 0.67 | 1.00 | 0.82 | 0.67 | 1.00 | 0.82 | 0.67 | 1.00 |
| 1I2DA | 0.58 | 0.33 | 1.00 | 0.82 | 0.67 | 1.00 | 0.58 | 0.33 | 1.00 | 1.00 | 1.00 | 1.00 |
| 1ITKA | 1.00 | 1.00 | 1.00 | 1.00 | 1.00 | 1.00 | 1.00 | 1.00 | 1.00 | 1.00 | 1.00 | 1.00 |
| 1IYKA | 0.82 | 0.67 | 1.00 | 1.00 | 1.00 | 1.00 | 1.00 | 1.00 | 1.00 | 1.00 | 1.00 | 1.00 |
| 1J3NA | 1.00 | 1.00 | 1.00 | 1.00 | 1.00 | 1.00 | 1.00 | 1.00 | 1.00 | 1.00 | 1.00 | 1.00 |
| 1JCZA | 0.71 | 0.50 | 1.00 | 0.71 | 0.50 | 1.00 | 0.71 | 0.50 | 1.00 | 0.71 | 0.50 | 1.00 |
| 1JNKA | 0.58 | 0.33 | 1.00 | 1.00 | 1.00 | 1.00 | 0.58 | 0.33 | 1.00 | 1.00 | 1.00 | 1.00 |
| 1JS4A | 0.87 | 0.75 | 1.00 | 0.87 | 0.75 | 1.00 | 0.50 | 0.25 | 1.00 | 0.87 | 0.75 | 1.00 |
| 1K2RA | 0.87 | 0.75 | 1.00 | 0.87 | 0.75 | 1.00 | 0.87 | 0.75 | 1.00 | 1.00 | 1.00 | 1.00 |
| 1K4LA | 0.82 | 0.67 | 1.00 | 1.00 | 1.00 | 1.00 | 1.00 | 1.00 | 1.00 | 1.00 | 1.00 | 1.00 |
| 1KFWA | 0.77 | 0.60 | 1.00 | 0.89 | 0.80 | 1.00 | 0.89 | 0.80 | 1.00 | 1.00 | 1.00 | 1.00 |
| 1KHBA | 0.58 | 0.33 | 1.00 | 0.82 | 0.67 | 1.00 | 0.58 | 0.33 | 1.00 | 0.82 | 0.67 | 1.00 |
| 1L8XA | 0.71 | 0.50 | 1.00 | 0.71 | 0.50 | 1.00 | 0.71 | 0.50 | 1.00 | 0.71 | 0.50 | 1.00 |
| 1LARA | 0.26 | 0.07 | 1.00 | 0.86 | 0.73 | 1.00 | 0.36 | 0.13 | 1.00 | 0.86 | 0.73 | 1.00 |
| 1LMEA | 0.91 | 0.83 | 1.00 | 0.96 | 0.92 | 1.00 | 1.00 | 1.00 | 1.00 | 1.00 | 1.00 | 1.00 |
| 1M56A | 1.00 | 1.00 | 1.00 | 1.00 | 1.00 | 1.00 | 0.71 | 1.00 | 0.50 | 1.00 | 1.00 | 1.00 |
| 1MGRA | 0.41 | 0.33 | 0.50 | 0.82 | 0.67 | 1.00 | 0.58 | 0.33 | 1.00 | 0.82 | 0.67 | 1.00 |
| 1MO0A | 0.78 | 0.61 | 1.00 | 0.75 | 0.56 | 1.00 | 0.82 | 0.67 | 1.00 | 0.82 | 0.67 | 1.00 |
| 1MZHA | 0.91 | 1.00 | 0.83 | 0.91 | 1.00 | 0.83 | 1.00 | 1.00 | 1.00 | 1.00 | 1.00 | 1.00 |
| 1N9BA | 0.78 | 0.62 | 1.00 | 1.00 | 1.00 | 1.00 | 0.92 | 0.85 | 1.00 | 1.00 | 1.00 | 1.00 |
| 1NDBA | 1.00 | 1.00 | 1.00 | 1.00 | 1.00 | 1.00 | 1.00 | 1.00 | 1.00 | 1.00 | 1.00 | 1.00 |
| 1NIDA | 0.76 | 1.00 | 0.57 | 0.76 | 1.00 | 0.57 | 0.76 | 1.00 | 0.57 | 0.71 | 1.00 | 0.50 |
| 1NMWA | 0.58 | 0.33 | 1.00 | 0.82 | 0.67 | 1.00 | 0.58 | 0.33 | 1.00 | 0.82 | 0.67 | 1.00 |
| 1O86A | 0.82 | 0.67 | 1.00 | 1.00 | 1.00 | 1.00 | 0.82 | 0.67 | 1.00 | 1.00 | 1.00 | 1.00 |
| 1OEPA | 0.87 | 0.75 | 1.00 | 1.00 | 1.00 | 1.00 | 1.00 | 1.00 | 1.00 | 1.00 | 1.00 | 1.00 |
| 1OJ4A | 0.82 | 0.67 | 1.00 | 0.82 | 0.67 | 1.00 | 0.82 | 0.67 | 1.00 | 0.82 | 0.67 | 1.00 |
| 1ONRA | 1.00 | 1.00 | 1.00 | 1.00 | 1.00 | 1.00 | 0.87 | 0.75 | 1.00 | 1.00 | 1.00 | 1.00 |
| 1P5HA | 0.77 | 1.00 | 0.60 | 0.77 | 1.00 | 0.60 | 0.77 | 1.00 | 0.60 | 0.77 | 1.00 | 0.60 |
| 1PEMA | 0.58 | 0.33 | 1.00 | 0.82 | 0.67 | 1.00 | 0.82 | 0.67 | 1.00 | 0.82 | 0.67 | 1.00 |
| 1PFKA | 0.50 | 0.25 | 1.00 | 0.71 | 0.50 | 1.00 | 0.50 | 0.25 | 1.00 | 0.71 | 0.50 | 1.00 |
| 1QHFA | 1.00 | 1.00 | 1.00 | 1.00 | 1.00 | 1.00 | 1.00 | 1.00 | 1.00 | 1.00 | 1.00 | 1.00 |
| 1QK1A | 0.58 | 0.33 | 1.00 | 0.71 | 0.50 | 1.00 | 0.47 | 0.33 | 0.67 | 0.82 | 0.67 | 1.00 |
| 1QPNA | 0.63 | 0.40 | 1.00 | 0.63 | 0.40 | 1.00 | 0.77 | 0.60 | 1.00 | 0.89 | 0.80 | 1.00 |
| 1QSGA | 1.00 | 1.00 | 1.00 | 1.00 | 1.00 | 1.00 | 1.00 | 1.00 | 1.00 | 1.00 | 1.00 | 1.00 |
| 1QWLA | 1.00 | 1.00 | 1.00 | 1.00 | 1.00 | 1.00 | 1.00 | 1.00 | 1.00 | 1.00 | 1.00 | 1.00 |
| 1QWOA | 1.00 | 1.00 | 1.00 | 1.00 | 1.00 | 1.00 | 1.00 | 1.00 | 1.00 | 1.00 | 1.00 | 1.00 |
| **1R1DA*** | 0.38 | 1.00 | 0.14 | 0.38 | 1.00 | 0.14 | 0.40 | 1.00 | 0.16 | 0.40 | 1.00 | **0.16** |
| 1RP1A | 0.71 | 0.50 | 1.00 | 0.71 | 0.50 | 1.00 | 0.71 | 0.50 | 1.00 | 0.71 | 0.50 | 1.00 |
| 1S1MA | 1.00 | 1.00 | 1.00 | 1.00 | 1.00 | 1.00 | 1.00 | 1.00 | 1.00 | 1.00 | 1.00 | 1.00 |
| 1S57A | 0.97 | 0.95 | 1.00 | 1.00 | 1.00 | 1.00 | 0.97 | 0.95 | 1.00 | 0.97 | 0.95 | 1.00 |
| 1T2AA | 1.00 | 1.00 | 1.00 | 1.00 | 1.00 | 1.00 | 1.00 | 1.00 | 1.00 | 1.00 | 1.00 | 1.00 |
| 1V8BA | 0.58 | 0.33 | 1.00 | 0.82 | 0.67 | 1.00 | 0.58 | 0.33 | 1.00 | 0.82 | 0.67 | 1.00 |
| 1VC4A | 0.82 | 0.67 | 1.00 | 1.00 | 1.00 | 1.00 | 1.00 | 1.00 | 1.00 | 1.00 | 1.00 | 1.00 |
| 1VDCA | 0.58 | 1.00 | 0.33 | 0.60 | 1.00 | 0.36 | 0.75 | 1.00 | 0.56 | 0.79 | 1.00 | 0.63 |
| 1VLCA | 0.93 | 0.86 | 1.00 | 1.00 | 1.00 | 1.00 | 0.93 | 0.86 | 1.00 | 1.00 | 1.00 | 1.00 |
| 1WE1A | 0.89 | 0.80 | 1.00 | 0.89 | 0.80 | 1.00 | 1.00 | 1.00 | 1.00 | 1.00 | 1.00 | 1.00 |
| 1WL4A | 0.87 | 1.00 | 0.75 | 0.87 | 1.00 | 0.75 | 0.87 | 1.00 | 0.75 | 0.87 | 1.00 | 0.75 |
| 1WO8A | 0.82 | 0.67 | 1.00 | 0.82 | 0.67 | 1.00 | 0.82 | 0.67 | 1.00 | 0.82 | 0.67 | 1.00 |
| 1XG2A | 1.00 | 1.00 | 1.00 | 1.00 | 1.00 | 1.00 | 1.00 | 1.00 | 1.00 | 1.00 | 1.00 | 1.00 |
| 1Y8GA | 0.41 | 0.17 | 1.00 | 0.58 | 0.33 | 1.00 | 0.58 | 0.33 | 1.00 | 0.71 | 0.50 | 1.00 |
| 1YISA | 0.71 | 0.50 | 1.00 | 0.71 | 0.50 | 1.00 | 0.71 | 0.50 | 1.00 | 0.71 | 0.50 | 1.00 |
| 1YRPA | 0.42 | 0.18 | 1.00 | 0.77 | 0.59 | 1.00 | 0.84 | 0.71 | 1.00 | 0.84 | 0.71 | 1.00 |
| 1YTMA | 0.63 | 0.40 | 1.00 | 0.89 | 0.80 | 1.00 | 0.63 | 0.40 | 1.00 | 0.77 | 0.60 | 1.00 |
| 1Z4RA | 0.82 | 0.67 | 1.00 | 1.00 | 1.00 | 1.00 | 0.82 | 0.67 | 1.00 | 1.00 | 1.00 | 1.00 |
| 1Z7WA | 1.00 | 1.00 | 1.00 | 1.00 | 1.00 | 1.00 | 1.00 | 1.00 | 1.00 | 1.00 | 1.00 | 1.00 |
| 1ZIOA | 0.77 | 0.60 | 1.00 | 0.84 | 0.70 | 1.00 | 0.84 | 0.70 | 1.00 | 0.89 | 0.80 | 1.00 |
| 1ZUWA | 0.61 | 0.38 | 1.00 | 0.79 | 0.63 | 1.00 | 0.79 | 0.63 | 1.00 | 1.00 | 1.00 | 1.00 |
| 2A6PA | 0.58 | 0.33 | 1.00 | 1.00 | 1.00 | 1.00 | 0.82 | 0.67 | 1.00 | 1.00 | 1.00 | 1.00 |
| 2AAZA | 0.82 | 0.67 | 1.00 | 1.00 | 1.00 | 1.00 | 1.00 | 1.00 | 1.00 | 1.00 | 1.00 | 1.00 |
| 2B7AA | 0.75 | 0.56 | 1.00 | 0.82 | 0.67 | 1.00 | 0.82 | 0.67 | 1.00 | 0.88 | 0.78 | 1.00 |
| 2BLTA | 0.87 | 0.75 | 1.00 | 1.00 | 1.00 | 1.00 | 1.00 | 1.00 | 1.00 | 1.00 | 1.00 | 1.00 |
| 2C07A | 0.87 | 0.75 | 1.00 | 0.87 | 0.75 | 1.00 | 0.87 | 0.75 | 1.00 | 0.87 | 0.75 | 1.00 |
| 2C2NA | 0.82 | 0.67 | 1.00 | 0.82 | 0.67 | 1.00 | 0.58 | 0.33 | 1.00 | 0.82 | 0.67 | 1.00 |
| 2DQ7X | 0.87 | 0.75 | 1.00 | 0.71 | 0.50 | 1.00 | 0.87 | 0.75 | 1.00 | 1.00 | 1.00 | 1.00 |
| 2EBDA | 1.00 | 1.00 | 1.00 | 1.00 | 1.00 | 1.00 | 1.00 | 1.00 | 1.00 | 1.00 | 1.00 | 1.00 |
| 2EHHA | 1.00 | 1.00 | 1.00 | 1.00 | 1.00 | 1.00 | 1.00 | 1.00 | 1.00 | 1.00 | 1.00 | 1.00 |
| 2EKCA | 0.58 | 0.33 | 1.00 | 0.82 | 0.67 | 1.00 | 0.58 | 0.33 | 1.00 | 0.58 | 0.33 | 1.00 |
| 2EP5A | 1.00 | 1.00 | 1.00 | 1.00 | 1.00 | 1.00 | 1.00 | 1.00 | 1.00 | 1.00 | 1.00 | 1.00 |
| 2EWDA | 0.58 | 0.33 | 1.00 | 0.78 | 0.61 | 1.00 | 0.53 | 0.28 | 1.00 | 0.71 | 0.50 | 1.00 |
| 2F57A | 0.82 | 0.67 | 1.00 | 1.00 | 1.00 | 1.00 | 1.00 | 1.00 | 1.00 | 1.00 | 1.00 | 1.00 |
| 2FEKA | 0.58 | 0.33 | 1.00 | 0.82 | 0.67 | 1.00 | 0.58 | 0.33 | 1.00 | 0.82 | 0.67 | 1.00 |
| 2GBCA | 1.00 | 1.00 | 1.00 | 0.89 | 1.00 | 0.80 | 1.00 | 1.00 | 1.00 | 0.89 | 1.00 | 0.80 |
| 2GFOA | 0.75 | 0.75 | 0.75 | 0.75 | 0.75 | 0.75 | 0.75 | 0.75 | 0.75 | 0.75 | 0.75 | 0.75 |
| 2GSAA | 0.82 | 0.67 | 1.00 | 1.00 | 1.00 | 1.00 | 0.82 | 0.67 | 1.00 | 1.00 | 1.00 | 1.00 |
| 2HB6A | 0.82 | 0.67 | 1.00 | 1.00 | 1.00 | 1.00 | 0.82 | 0.67 | 1.00 | 0.82 | 0.67 | 1.00 |
| 2HCYA | 0.58 | 0.33 | 1.00 | 0.82 | 0.67 | 1.00 | 0.82 | 0.67 | 1.00 | 0.82 | 0.67 | 1.00 |
| 2HFSA | 0.82 | 0.67 | 1.00 | 0.82 | 0.67 | 1.00 | 0.58 | 0.33 | 1.00 | 0.82 | 0.67 | 1.00 |
| **2HIHA*** | 0.38 | 1.00 | 0.14 | 0.38 | 1.00 | 0.14 | 0.40 | 1.00 | 0.16 | 0.40 | 1.00 | **0.16** |
| 2HS6A | 1.00 | 1.00 | 1.00 | 1.00 | 1.00 | 1.00 | 1.00 | 1.00 | 1.00 | 1.00 | 1.00 | 1.00 |
| 2I6UA | 0.63 | 0.40 | 1.00 | 0.77 | 0.60 | 1.00 | 0.77 | 0.60 | 1.00 | 0.77 | 0.60 | 1.00 |
| 2IV0A | 0.67 | 0.67 | 0.67 | 1.00 | 1.00 | 1.00 | 0.61 | 0.50 | 0.75 | 0.91 | 0.83 | 1.00 |
| 2NACA | 0.71 | 0.50 | 1.00 | 0.71 | 0.50 | 1.00 | 0.71 | 0.50 | 1.00 | 0.71 | 0.50 | 1.00 |
| 2OHCA | 1.00 | 1.00 | 1.00 | 1.00 | 1.00 | 1.00 | 1.00 | 1.00 | 1.00 | 1.00 | 1.00 | 1.00 |
| 2OK7A | 1.00 | 1.00 | 1.00 | 1.00 | 1.00 | 1.00 | 1.00 | 1.00 | 1.00 | 1.00 | 1.00 | 1.00 |
| 2PA6A | 0.82 | 0.67 | 1.00 | 0.82 | 0.67 | 1.00 | 1.00 | 1.00 | 1.00 | 1.00 | 1.00 | 1.00 |
| 2PGDA | 0.89 | 0.80 | 1.00 | 0.91 | 1.00 | 0.83 | 0.80 | 0.80 | 0.80 | 0.79 | 1.00 | 0.63 |
| 2Q3EA | 0.82 | 0.67 | 1.00 | 0.82 | 0.67 | 1.00 | 0.82 | 0.67 | 1.00 | 0.82 | 0.67 | 1.00 |
| 2Q8NA | 0.65 | 0.43 | 1.00 | 0.85 | 0.71 | 1.00 | 0.76 | 0.86 | 0.67 | 0.93 | 0.86 | 1.00 |
| 2QFLA | 0.50 | 0.25 | 1.00 | 0.71 | 0.50 | 1.00 | 0.50 | 0.25 | 1.00 | 0.87 | 0.75 | 1.00 |
| 2QT6A | 1.00 | 1.00 | 1.00 | 1.00 | 1.00 | 1.00 | 1.00 | 1.00 | 1.00 | 1.00 | 1.00 | 1.00 |
| 2RD5A | 0.91 | 0.83 | 1.00 | 0.91 | 0.83 | 1.00 | 0.83 | 0.83 | 0.83 | 0.91 | 0.83 | 1.00 |
| 2REIA | 0.31 | 0.10 | 1.00 | 0.90 | 0.81 | 1.00 | 0.76 | 0.57 | 1.00 | 0.95 | 0.90 | 1.00 |
| 2VGBA | 0.53 | 0.29 | 1.00 | 0.53 | 0.29 | 1.00 | 0.65 | 0.43 | 1.00 | 0.76 | 0.57 | 1.00 |
| 2YXXA | 0.82 | 0.67 | 1.00 | 1.00 | 1.00 | 1.00 | 1.00 | 1.00 | 1.00 | 1.00 | 1.00 | 1.00 |
| **mean** | **0.79** | **0.71** | **0.95** | **0.88** | **0.83** | **0.96** | **0.82** | **0.75** | **0.94** | **0.90** | **0.86** | **0.96** |
| **Std** | **0.18** | **0.27** | **0.14** | **0.13** | **0.20** | **0.14** | **0.17** | **0.25** | **0.14** | **0.12** | **0.18** | **0.12** |
