# Supplementary material for: CMASA: an accurate algorithm for detecting local protein structural similarity and its application to enzyme catalytic site annotation
Source: BMC Bioinformatics. 2010 Aug 27;11:439. doi: 10.1186/1471-2105-11-439 (PMC2936402; doi:10.1186/1471-2105-11-439)
Supplement: Additional file 3 — Table S3: Predicted active sites with P-value < 1.0 × 10^-4^ and their best matching MCT CSA. Only showed the predicted structures deposited before 2008. [file 1471-2105-11-439-S3.DOC]

| **Number** | **PDBid** | **Matched MCT CSA** | **Predicted active sites** | **MCT CSA active sites** | **CMAD** | **RMSD** | **P-value** |
| --- | --- | --- | --- | --- | --- | --- | --- |
| 1 | 1SR4B | 1AKOA | N33-D199-N201-D238-H274 | N7-D151-N153-D229-H259 | 0.139 | 0.147 | 0.00E+00 |
| 2 | 2EI9A | 2JC4A | N17-Y99-D130-N132-D190-H213 | N7-Y105-D146-N148-D217-H247 | 0.251 | 0.426 | 0.00E+00 |
| 3 | 2F1NA | 1AKOA | N11-D174-N176-D211-H243 | N7-D151-N153-D229-H259 | 0.105 | 0.217 | 0.00E+00 |
| 4 | 1P3CA | 1B0FA | H47-D97-G169-S171-G172 | H57-D102-G193-S195-G196 | 0.155 | 0.284 | 1.11E-16 |
| 5 | 1WXRA | 1AUTC | H73-D101-G205-S207-G208 | H57-D102-G193-S195-G196 | 0.246 | 0.292 | 3.33E-16 |
| 6 | 1ZYOA | 1A0HB | H181-D216-G282-S284-G285 | H363-D419-G523-S525-G526 | 0.205 | 0.311 | 2.78E-15 |
| 7 | 2B30A | 1F5SA | D33-F34-D35-G69-K225-D252 | D11-F12-D13-G100-K144-D171 | 0.333 | 0.503 | 9.99E-15 |
| 8 | 1LBSA | 1BS9A | T40-S105-Q106-D187-H224 | T13-S90-Q91-D175-H187 | 0.174 | 0.413 | 2.02E-14 |
| 9 | 1MBMA | 1AU8A | H39-D65-G118-S120-G121 | H57-D102-G193-S195-G196 | 0.143 | 0.378 | 8.14E-14 |
| 10 | 1NOFA | 2OSWA | R81-N164-E165-H230-Y232-E253 | R93-N232-E233-H304-Y306-E351 | 0.44 | 0.685 | 3.17E-13 |
| 11 | 1ARBA | 1XX9A | H57-D113-G192-S194-G195-P197 | H57-D102-G193-S195-G196-P198 | 0.374 | 0.57 | 3.44E-13 |
| 12 | 1P49A | 1HDHA | R79-K134-H136-H290-D342-K368 | R55-K113-H115-H211-D317-K375 | 0.522 | 0.652 | 3.50E-13 |
| 13 | 1XKGA | 1A6RA | Q108-A114-H250-N270 | Q67-A73-H369-N392 | 0.163 | 0.248 | 5.45E-13 |
| 14 | 1VLQA | 1L7AA | S188-Q189-D274-H303 | S181-Q182-D269-H298 | 0.258 | 0.287 | 5.62E-13 |
| 15 | 1DT2A | 1OP8A | H65-D114-G184-S186-G187 | H57-D102-G193-S195-G196 | 0.322 | 0.445 | 9.63E-13 |
| 16 | 1IV8A | 2GUYA | H90-R226-D228-E255-H442-D443 | H122-R204-D206-E230-H296-D297 | 0.457 | 0.672 | 1.20E-12 |
| 17 | 3B5QA | 1HDHA | R68-K117-H119-H180-D283-K296 | R55-K113-H115-H211-D317-K375 | 0.41 | 0.656 | 1.21E-12 |
| 18 | 2NW0A | 1OBAA | D6-D90-E92-D171 | D10-D92-E94-D182 | 0.182 | 0.276 | 1.27E-11 |
| 19 | 1YWFA | 2IMGA | D82-C160-R166-T167 | D65-C95-R101-T102 | 0.279 | 0.344 | 1.44E-11 |
| 20 | 1WDUA | 2JC4A | N29-Y126-D157-N159-D211-H237 | N7-Y105-D146-N148-D217-H247 | 0.401 | 0.796 | 3.08E-11 |
| 21 | 1LVBA | 2BHGA | H46-D81-A151-S168 | H46-D84-A163-S182 | 0.329 | 0.364 | 3.54E-11 |
| 22 | 1JFRA | 1AZWA | S131-D177-H209 | S110-D266-H294 | 0.142 | 0.139 | 6.21E-11 |
| 23 | 2F7VA | 1CG2A | H72-D103-E130-E131-E155-H340 | H112-D141-E175-E176-E200-H385 | 0.435 | 0.786 | 6.98E-11 |
| 24 | 2QRUA | 1A88A | S102-D219-H247 | S96-D226-H255 | 0.124 | 0.177 | 4.12E-10 |
| 25 | 2CL2A | 1AJOA | E115-D117-E120 | E193-D195-E197 | 0.185 | 0.191 | 4.13E-10 |
| 26 | 2QJWA | 1A88A | S81-D129-H155 | S96-D226-H255 | 0.154 | 0.169 | 6.70E-10 |
| 27 | 2VEOA | 1VE6A | S184-D334-H366 | S445-D524-H556 | 0.152 | 0.209 | 1.07E-09 |
| 28 | 2QS9A | 1A88A | S75-D138-H165 | S96-D226-H255 | 0.131 | 0.197 | 2.44E-09 |
| 29 | 1XJEA | 1PEMA | C134-N320-C322-E324-C333 | C178-N386-C388-E390-C415 | 0.647 | 1.002 | 2.49E-09 |
| 30 | 1KXAA | 1RGQA | H141-D163-G213-S215 | H60-D84-G140-S142 | 0.339 | 0.448 | 2.94E-09 |
| 31 | 2R8BA | 1JKMA | S147-D197-H228 | S202-D308-H338 | 0.271 | 0.24 | 6.32E-09 |
| 32 | 2FX5A | 1BS9A | T58-S126-Q127-D176-H206 | T13-S90-Q91-D175-H187 | 0.449 | 0.762 | 8.02E-09 |
| 33 | 1UKFA | 2GFOA | N93-C98-H212-D227 | N781-C786-H1067-D1084 | 0.468 | 0.534 | 8.17E-09 |
| 34 | 1YN9A | 2CFVA | H118-C119-R125-T126 | H1238-C1239-R1245-T1246 | 0.361 | 0.548 | 9.75E-09 |
| 35 | 1DYLA | 1SGFG | H145-D167-G217-S219-G220 | H57-D102-G193-S195-G196 | 0.417 | 0.699 | 1.15E-08 |
| 36 | 1UXOA | 1A88A | S71-D137-H164 | S96-D226-H255 | 0.198 | 0.229 | 1.62E-08 |
| 37 | 1FLCA | 1FXWF | S57-G85-N117-D352-H355 | S48-G75-N105-D193-H196 | 0.523 | 0.789 | 1.75E-08 |
| 38 | 1TIAA | 2HIHA | S145-D199-H259 | S124-D314-H355 | 0.226 | 0.269 | 1.81E-08 |
| 39 | 1IMJA | 2HIHA | S111-D162-H188 | S124-D314-H355 | 0.181 | 0.268 | 1.86E-08 |
| 40 | 1XRIA | 2CFVA | H149-C150-R156-T157 | H1238-C1239-R1245-T1246 | 0.233 | 0.61 | 2.02E-08 |
| 41 | 2BF6A | 1SLLA | D291-E539-Y655 | D318-E595-Y713 | 0.192 | 0.237 | 2.11E-08 |
| 42 | 1YCDA | 1E5TA | S110-D183-H218 | S554-D641-H680 | 0.236 | 0.246 | 2.28E-08 |
| 43 | 1VKHA | 1YSCA | S110-D211-H243 | S146-D338-H397 | 0.26 | 0.264 | 2.59E-08 |
| 44 | 3B5EA | 1A88A | S118-D168-H198 | S96-D226-H255 | 0.216 | 0.274 | 2.72E-08 |
| 45 | 1DT3A | 1E5TA | S146-D201-H258 | S554-D641-H680 | 0.224 | 0.275 | 3.08E-08 |
| 46 | 2IY9A | 1MEEA | D52-H89-N183-S272 | D32-H64-N155-S221 | 0.493 | 0.612 | 3.19E-08 |
| 47 | 1ZY7A | 1VQ2A | E396-S449-C451 | E106-S130-C132 | 0.216 | 0.291 | 3.58E-08 |
| 48 | 2Z8XA | 1VE6A | S207-D255-H313 | S445-D524-H556 | 0.192 | 0.282 | 4.89E-08 |
| 49 | 2PKEA | 2HSZA | D18-D20-T133-K162 | D10-D12-T118-K151 | 0.397 | 0.608 | 5.65E-08 |
| 50 | 1JJFA | 1R4ZA | I90-S172-M173-D230-H260 | I12-S77-M78-D133-H156 | 0.517 | 0.94 | 7.41E-08 |
| 51 | 1EP5B | 1XX9A | H152-D174-G224-S226-G227-P229 | H57-D102-G193-S195-G196-P198 | 0.686 | 1.003 | 7.45E-08 |
| 52 | 2QUAA | 1VE6A | S207-D256-H314 | S445-D524-H556 | 0.205 | 0.288 | 7.93E-08 |
| 53 | 2FUKA | 1A88A | S119-D166-H195 | S96-D226-H255 | 0.303 | 0.306 | 8.67E-08 |
| 54 | 1PV1A | 1JKMA | S161-D241-H276 | S202-D308-H338 | 0.173 | 0.289 | 8.74E-08 |
| 55 | 1LGYA | 1JKMA | S145-D204-H257 | S202-D308-H338 | 0.334 | 0.313 | 9.94E-08 |
| 56 | 1W30A | 1P0CA | S127-S130-I85 | S1048-S1051-I1057 | 0.274 | 0.296 | 2.25E-07 |
| 57 | 2BKAA | 1EQ2A | S132-Y143-K147 | S116-Y140-K144 | 0.314 | 0.336 | 2.39E-07 |
| 58 | 1WVUA | 1CNSA | E147-E156-S190 | E67-E89-S120 | 0.25 | 0.356 | 2.73E-07 |
| 59 | 3BDIA | 2HIHA | S107-D157-H185 | S124-D314-H355 | 0.181 | 0.357 | 4.08E-07 |
| 60 | 2I3DA | 2HIHA | S108-D157-H190 | S124-D314-H355 | 0.221 | 0.373 | 4.41E-07 |
| 61 | 1USWA | 2HIHA | S133-D194-H247 | S124-D314-H355 | 0.264 | 0.388 | 6.72E-07 |
| 62 | 2UZ0A | 1JKMA | S120-D202-H231 | S202-D308-H338 | 0.317 | 0.417 | 8.79E-07 |
| 63 | 2B1MA | 1YALA | Q20-H168-N188 | Q19-H159-N179 | 0.33 | 0.445 | 1.02E-06 |
| 64 | 1THTA | 2PL5A | S114-D211-H241 | S153-D311-H344 | 0.282 | 0.387 | 1.24E-06 |
| 65 | 1ZZWA | 1OHCA | D377-C408-R414 | D287-C314-R320 | 0.402 | 0.496 | 1.32E-06 |
| 66 | 2J2CA | 2HSZA | D52-D54-T249-K292 | D10-D12-T118-K151 | 0.659 | 0.689 | 1.36E-06 |
| 67 | 2JBWA | 1YSCA | S217-D300-H329 | S146-D338-H397 | 0.376 | 0.432 | 1.72E-06 |
| 68 | 1DILA | 1SLLA | D62-E231-Y342 | D318-E595-Y713 | 0.516 | 0.438 | 1.90E-06 |
| 69 | 1NNHA | 12ASA | D47-R99-Q116 | D46-R100-Q116 | 0.422 | 0.465 | 2.00E-06 |
| 70 | 1KQHA | 2DD4C | C23-S24-C25 | C131-S132-C133 | 0.213 | 0.28 | 2.09E-06 |
| 71 | 2RAEA | 1MPYA | H72-H111-Y171 | H199-H246-Y255 | 0.346 | 0.58 | 2.09E-06 |
| 72 | 2QTQA | 1JS4A | D102-D106-E191 | D55-D58-E424 | 0.3 | 0.388 | 2.75E-06 |
| 73 | 2ESBA | 1OHCA | D73-C104-R110 | D287-C314-R320 | 0.387 | 0.516 | 2.76E-06 |
| 74 | 2FMUA | 1UJMA | S131-S132-Y143-K147 | S132-S133-Y177-K181 | 0.607 | 0.77 | 3.04E-06 |
| 75 | 2CB9A | 1KEZA | S84-A85-D111-H201 | S142-A143-D169-H259 | 0.5 | 0.823 | 3.50E-06 |
| 76 | 2H1IA | 1R1DA | S103-D153-H184 | S93-D192-H222 | 0.582 | 0.482 | 3.65E-06 |
| 77 | 2V6EA | 2B9SA | R275-R383-H416 | R314-R410-H453 | 0.346 | 0.485 | 3.65E-06 |
| 78 | 1S24A | 1X5DA | C10-G11-H12-C7 | C37-G38-H39-C40 | 0.474 | 0.768 | 3.99E-06 |
| 79 | 2IQTA | 1ADOA | D20-E176-K211 | D33-E187-K229 | 0.269 | 0.42 | 4.14E-06 |
| 80 | 1SFRA | 1C2BA | S126-E230-H262 | S203-E334-H447 | 0.425 | 0.562 | 4.82E-06 |
| 81 | 2NT2A | 1J4XA | D361-S392-R398 | D92-S124-R130 | 0.427 | 0.446 | 5.03E-06 |
| 82 | 1T9ZA | 2HSZA | D96-D98-T152-K190 | D10-D12-T118-K151 | 0.342 | 0.809 | 5.12E-06 |
| 83 | 2HHLA | 2HSZA | D34-D36-T90-K128 | D10-D12-T118-K151 | 0.313 | 0.8 | 5.67E-06 |
| 84 | 2GP3A | 1AHJA | C306-S307-C308 | C113-S114-C115 | 0.37 | 0.637 | 6.44E-06 |
| 85 | 1BIKA | 2PPTA | C59-G33-P34-C35 | C73-G74-P75-C76 | 0.603 | 0.711 | 6.80E-06 |
| 86 | 2HNKA | 2IX5A | S74-A79-E66 | S287-A292-E408 | 0.368 | 0.431 | 6.96E-06 |
| 87 | 2DMIA | 1X5DA | C86-G87-H88-C83 | C37-G38-H39-C40 | 0.769 | 0.852 | 7.26E-06 |
| 88 | 1GZUA | 1BOOA | S49-P50-D83 | S53-P54-D96 | 0.559 | 0.52 | 7.78E-06 |
| 89 | 1QO7A | 2E3JA | D192-D348-H374 | D104-D302-H333 | 0.399 | 0.546 | 7.78E-06 |
| 90 | 1HUXA | 2CF5A | H98-T99-H112 | H48-T49-H52 | 0.405 | 0.609 | 8.34E-06 |
| 91 | 1ZR0B | 2PPTA | C38-G12-P13-C14 | C73-G74-P75-C76 | 0.634 | 0.759 | 9.09E-06 |
| 92 | 2Q5EA | 2HSZA | D107-D109-T163-K201 | D10-D12-T118-K151 | 0.462 | 0.835 | 9.36E-06 |
| 93 | 2J6AA | 1X5DA | C115-G116-H117-C112 | C37-G38-H39-C40 | 0.522 | 0.958 | 9.73E-06 |
| 94 | 1FPZA | 1A5YA | D110-R146-S147 | D181-R221-S222 | 0.215 | 0.48 | 1.04E-05 |
| 95 | 1VJVA | 1PADA | C118-H447-N465 | C25-H159-N175 | 0.226 | 0.645 | 1.09E-05 |
| 96 | 2A3LA | 2ADMA | N625-P626-Y631 | N105-P106-Y108 | 0.562 | 0.528 | 1.10E-05 |
| 97 | 1X2GA | 1J7GA | Q238-F235-T234 | Q78-F79-T80 | 0.482 | 0.53 | 1.15E-05 |
| 98 | 2FE8A | 1GX3A | C112-H273-D287 | C70-H110-D127 | 0.537 | 0.6 | 1.25E-05 |
| 99 | 1A5TA | 1X5DA | C59-G60-H61-C62 | C37-G38-H39-C40 | 0.464 | 1.003 | 1.29E-05 |
| 100 | 1OTVA | 1EQ2A | S143-Y128-K214 | S116-Y140-K144 | 0.531 | 0.563 | 1.32E-05 |
| 101 | 1YFUA | 1X5DA | C128-G129-H130-C125 | C37-G38-H39-C40 | 0.495 | 0.918 | 1.34E-05 |
| 102 | 2DXPA | 1YGRA | D69-S96-R102 | D796-S828-R834 | 0.499 | 0.525 | 1.34E-05 |
| 103 | 2ETAA | 1CVRA | E173-H165-G164-C157 | E152-H211-G212-C244 | 0.63 | 0.913 | 1.41E-05 |
| 104 | 2O56A | 1X8BA | D8-K29-S37-N31 | D426-K428-S430-N431 | 0.725 | 0.892 | 1.42E-05 |
| 105 | 1MEC3 | 1AHJA | C86-S87-C88 | C113-S114-C115 | 0.54 | 0.689 | 1.44E-05 |
| 106 | 1HSJA | 2NPXA | H412-S430-C429-R415 | H10-S41-C42-R303 | 0.777 | 1.384 | 1.46E-05 |
| 107 | 1CL7H | 2TMDA | Y47-H35-D95 | Y169-H172-D267 | 0.328 | 0.595 | 1.53E-05 |
| 108 | 1UB7A | 1BI5A | F155-H246-N276 | F215-H303-N336 | 0.664 | 0.676 | 1.53E-05 |
| 109 | 1G7RA | 1J7GA | Q153-F150-T149 | Q78-F79-T80 | 0.67 | 0.595 | 1.68E-05 |
| 110 | 1JS3A | 1C4KA | H192-D271-K303 | H223-D316-K355 | 0.544 | 0.607 | 1.75E-05 |
| 111 | 1GVYA | 1STCE | D150-K153-T160 | D166-K168-T201 | 0.466 | 0.534 | 1.82E-05 |
| 112 | 1TF1A | 1JYSA | D122-A68-F162 | D197-A199-F210 | 0.664 | 0.571 | 1.90E-05 |
| 113 | 1NX9A | 1UK7A | A205-D338-H370 | A103-D224-H252 | 0.357 | 0.509 | 1.92E-05 |
| 114 | 2C1GA | 1ZSYA | S62-N65-Y80 | S69-N72-Y78 | 0.485 | 0.546 | 1.99E-05 |
| 115 | 1JFXA | 1OBAA | D9-D98-E100-D198 | D10-D92-E94-D182 | 0.775 | 1.034 | 2.31E-05 |
| 116 | 1AAZA | 1VDCA | C14-C17-D18 | C135-C138-D139 | 0.282 | 0.639 | 2.37E-05 |
| 117 | 2Q14A | 1OKGA | D96-H99-S103 | D61-H75-S255 | 0.567 | 0.546 | 2.58E-05 |
| 118 | 2E0TA | 1OHCA | D120-C152-R158 | D287-C314-R320 | 0.482 | 0.661 | 2.69E-05 |
| 119 | 1I9BA | 1T90A | N90-L199-C123 | N153-L250-C284 | 0.533 | 0.644 | 2.74E-05 |
| 120 | 2HNHA | 1PIXA | V72-G137-I463-A438 | V151-G194-I417-A457 | 0.689 | 0.836 | 2.74E-05 |
| 121 | 1MZ5A | 1EUUA | D60-E231-Y343 | D92-E260-Y370 | 0.262 | 0.613 | 2.88E-05 |
| 122 | 2Z1DA | 2PPTA | C325-G352-P353-C354 | C73-G74-P75-C76 | 0.736 | 0.99 | 2.96E-05 |
| 123 | 2HLKA | 2HSZA | D64-D66-T124-K161 | D10-D12-T118-K151 | 0.51 | 0.862 | 2.99E-05 |
| 124 | 1UFOA | 1QJ4A | S113-D183-H217 | S80-D207-H235 | 0.232 | 0.722 | 3.13E-05 |
| 125 | 2IC8A | 1XZWA | H150-A253-H254 | H201-A294-H295 | 0.388 | 0.656 | 3.21E-05 |
| 126 | 1WXNA | 2DD4C | C4-S5-C6 | C131-S132-C133 | 0.266 | 0.431 | 3.28E-05 |
| 127 | 1CLIA | 2PCQA | Y194-N299-K70 | Y124-N129-K152 | 0.419 | 0.663 | 3.32E-05 |
| 128 | 1ANTL | 1AHJA | C247-S248-C430 | C113-S114-C115 | 0.547 | 0.762 | 3.34E-05 |
| 129 | 3BDVA | 1JKMA | S81-D135-H162 | S202-D308-H338 | 0.466 | 0.582 | 3.40E-05 |
| 130 | 1JI3A | 2PL5A | S113-D317-H358 | S153-D311-H344 | 0.401 | 0.585 | 3.48E-05 |
| 131 | 1A0OA | 1C17M | S15-R19-N23 | S206-R210-N214 | 0.51 | 0.642 | 3.56E-05 |
| 132 | 1SVVA | 1C4KA | H100-D183-K216 | H223-D316-K355 | 0.391 | 0.607 | 3.75E-05 |
| 133 | 1WUUA | 1J7GA | Q155-F152-T151 | Q78-F79-T80 | 0.615 | 0.645 | 3.88E-05 |
| 134 | 2BTWA | 1GX3A | C70-H183-D201 | C70-H110-D127 | 0.786 | 0.936 | 3.89E-05 |
| 135 | 1TGLA | 2PL5A | S144-D203-H257 | S153-D311-H344 | 0.244 | 0.609 | 3.95E-05 |
| 136 | 2D42A | 1T7DA | S230-S87-K130 | S88-S90-K145 | 0.494 | 0.635 | 3.99E-05 |
| 137 | 1VIOA | 1MX1A | S118-E52-H96 | S1221-E1354-H1468 | 0.36 | 0.592 | 4.27E-05 |
| 138 | 1MR5A | 1EUUA | D59-E230-Y342 | D92-E260-Y370 | 0.6 | 0.632 | 4.38E-05 |
| 139 | 2HCMA | 1OHCA | D64-C95-R101 | D287-C314-R320 | 0.434 | 0.631 | 4.39E-05 |
| 140 | 2B7OA | 1C17M | S167-R171-N175 | S206-R210-N214 | 0.234 | 0.615 | 4.46E-05 |
| 141 | 1OGMX | 2CF5A | H8-T7-H53 | H48-T49-H52 | 0.234 | 0.707 | 4.85E-05 |
| 142 | 2CW6A | 1AOZA | H235-C234-H233 | H506-C507-H508 | 0.537 | 0.882 | 4.90E-05 |
| 143 | 1FAKI | 2PPTA | C38-G12-P13-C14 | C73-G74-P75-C76 | 0.675 | 0.822 | 5.06E-05 |
| 144 | 2O6XA | 1VDCA | C195-C154-D153 | C135-C138-D139 | 0.388 | 0.769 | 5.11E-05 |
| 145 | 1X3WA | 1GX3A | C191-H218-D235 | C70-H110-D127 | 0.79 | 0.658 | 5.15E-05 |
| 146 | 1YZBA | 1CQDA | Q9-C14-H119-N134 | Q21-C27-H161-N181 | 0.665 | 1.063 | 5.21E-05 |
| 147 | 1AALA | 2PPTA | C38-G12-P13-C14 | C73-G74-P75-C76 | 0.691 | 0.862 | 5.26E-05 |
| 148 | 2G6ZA | 1J4XA | D232-S263-R269 | D92-S124-R130 | 0.565 | 0.604 | 5.58E-05 |
| 149 | 2P4DA | 1OHCA | D79-C110-R116 | D287-C314-R320 | 0.491 | 0.704 | 5.79E-05 |
| 150 | 1AAPA | 2PPTA | C38-G12-P13-C14 | C73-G74-P75-C76 | 0.674 | 0.845 | 6.04E-05 |
| 151 | 1NCGA | 2IX5A | S26-A78-E89 | S287-A292-E408 | 0.611 | 0.644 | 6.31E-05 |
| 152 | 2FUG3 | 1AHJA | C48-S82-C83 | C113-S114-C115 | 0.485 | 0.834 | 6.38E-05 |
| 153 | 2GQTA | 1ECFB | C198-N233-G209-S96 | C1-N101-G102-S345 | 0.567 | 1.198 | 6.72E-05 |
| 154 | 2DDIA | 2PPTA | C43-G17-P18-C19 | C73-G74-P75-C76 | 0.702 | 0.852 | 7.08E-05 |
| 155 | 2NX9A | 1AOZA | H207-C208-H209 | H506-C507-H508 | 0.649 | 0.91 | 7.10E-05 |
| 156 | 1BM3H | 1HZFA | C22-Q6-A126 | C991-Q994-A1292 | 0.841 | 0.716 | 8.07E-05 |
| 157 | 3B8FA | 1UAQA | E54-S87-C89 | E64-S89-C91 | 0.577 | 0.632 | 8.14E-05 |
| 158 | 2NYTA | 1VQ2A | E100-S126-C128 | E106-S130-C132 | 0.619 | 0.674 | 8.33E-05 |
| 159 | 1WA9A | 1J7GA | Q289-F286-T285 | Q78-F79-T80 | 0.739 | 0.656 | 8.36E-05 |
| 160 | 1JWQA | 2PTHA | N30-H10-D14 | N10-H20-D93 | 0.529 | 0.694 | 8.75E-05 |
| 161 | 1VRDA | 1STCE | D9-K455-T457 | D166-K168-T201 | 0.808 | 0.617 | 8.90E-05 |
| 162 | 1OHTA | 1VDCA | C50-C56-D55 | C135-C138-D139 | 0.495 | 0.793 | 8.91E-05 |
| 163 | 1YZ4A | 1J4XA | D57-S88-R94 | D92-S124-R130 | 0.709 | 0.663 | 9.30E-05 |
| 164 | 1O6UA | 1X8BA | D310-K376-S281-N378 | D426-K428-S430-N431 | 0.787 | 0.99 | 9.44E-05 |
| 165 | 2Q9RA | 2IX5A | S123-A128-E157 | S287-A292-E408 | 0.384 | 0.56 | 9.71E-05 |
| 166 | 2BTUA | 2PCQA | Y191-N298-K67 | Y124-N129-K152 | 0.844 | 0.815 | 9.78E-05 |
