# Supplementary figures and images for: CMASA: an accurate algorithm for detecting local protein structural similarity and its application to enzyme catalytic site annotation
Source: BMC Bioinformatics. 2010 Aug 27;11:439. doi: 10.1186/1471-2105-11-439 (PMC2936402; doi:10.1186/1471-2105-11-439)

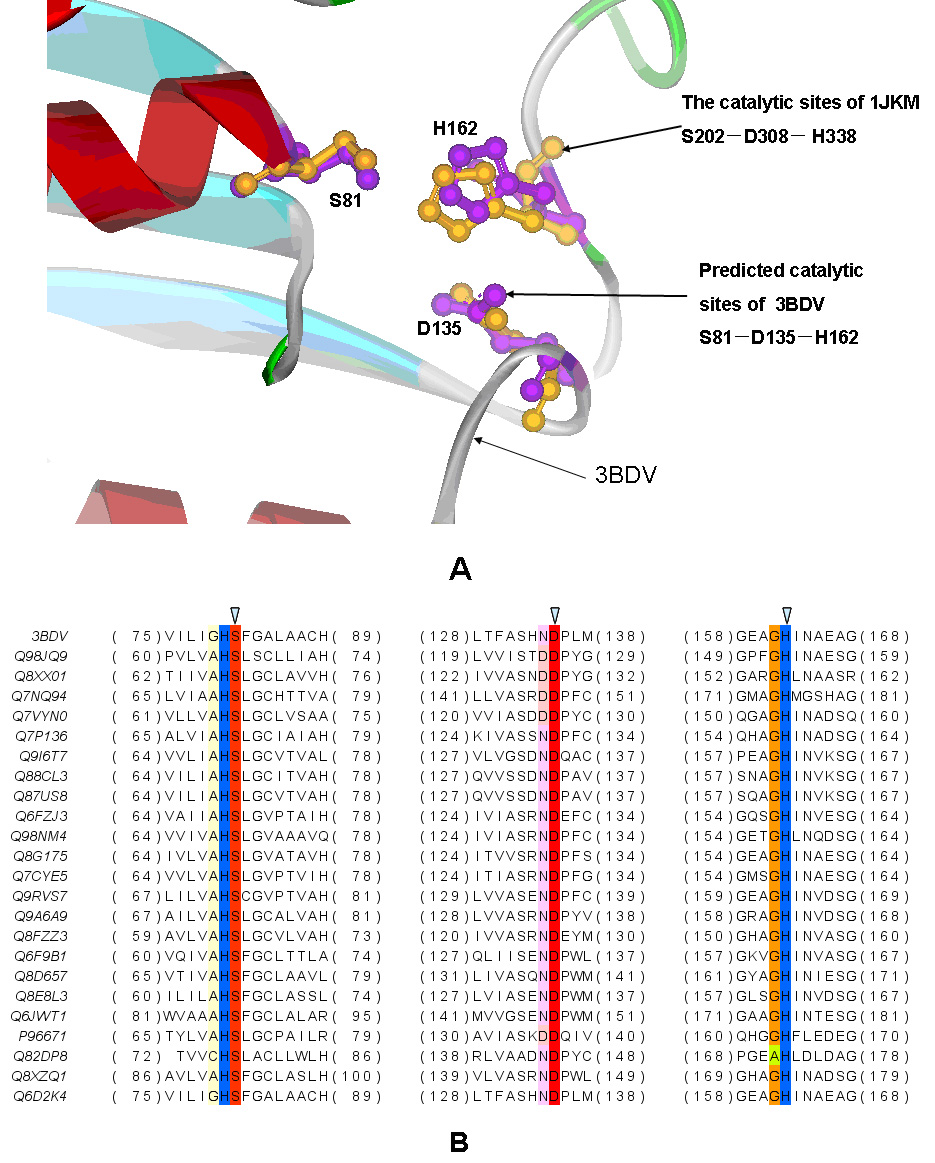

Supplement: Additional file 4 — Figure S1: Predicting 3BDV catalytic sites using CMASA. A: The CMASA superposition result. The best hit, a serine hydrolase (PDBid:1JKM) with the catalytic sites of S202-D303-H338, is shown. The predicted 3BDV catalytic sites (S81, D135 and H162) are labelled. B: the sequence alignment of the DUF123 family, these sequences are directly from Pfam[29] seed sequences. The predicted catalytic sites are labelled by inverted triangles. [file 1471-2105-11-439-S4.JPEG]

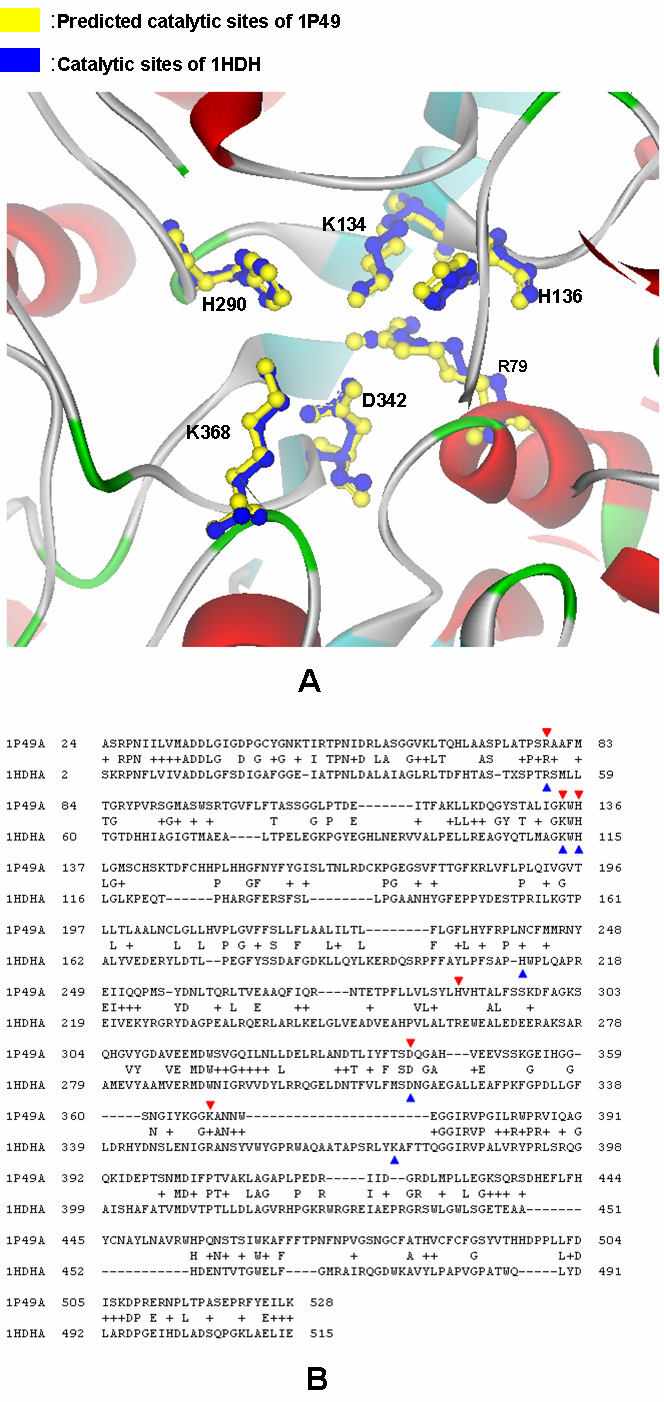

Supplement: Additional file 5 — Figure S2: Predicting the catalytic sites of human placental estrone sulfatase (PDBid:1P49) using CMASA. A: the CMASA superposition result. The best hit, an arylsulfatase (PDBid: 1HDH), which hold the catalytic sites of R55-K113-H115-H211-D317-K375, is shown. The predicted 1P49 catalytic sites (R79, K134, H136, H290, D342 and K368) are labelled. B: PSI-BLAST result between 1P49 and 1HDH. The predicted 1P49 catalytic sites and the 1HDH catalytic sites are labelled as inverted red and blue triangles. [file 1471-2105-11-439-S5.JPEG]
